# Exploring consensus in 21st century projections of climatically suitable areas for African vertebrates

**DOI:** 10.1111/j.1365-2486.2011.02605.x

**Published:** 2011-12-30

**Authors:** Raquel A Garcia, Neil D Burgess, Mar Cabeza, Carsten Rahbek, Miguel B Araújo

**Affiliations:** * Department of Biodiversity and Evolutionary Biology, National Museum of Natural Sciences, CSICCalle José Gutierrez Abascal, 28006, Madrid, Spain; † Center for Macroecology, Evolution and Climate, Department of Biology, University of Copenhagen2100, Copenhagen, Denmark; ‡ Rui Nabeiro Biodiversity Chair, University of Évora, CIBIOLargo dos Colegiais, 7000, Évora, Portugal; § WWF US Conservation Science Program1250 24th Street NW, Washington, DC, USA; ¶ Metapopulation Research Group, Department of Biosciences, University of HelsinkiFIN-00014, Helsinki, Finland

**Keywords:** Africa, bioclimatic envelope model, climate change, consensus, ensemble forecasting, nonanalogue climate, uncertainty, vertebrates

## Abstract

Africa is predicted to be highly vulnerable to 21st century climatic changes. Assessing the impacts of these changes on Africa's biodiversity is, however, plagued by uncertainties, and markedly different results can be obtained from alternative bioclimatic envelope models or future climate projections. Using an ensemble forecasting framework, we examine projections of future shifts in climatic suitability, and their methodological uncertainties, for over 2500 species of mammals, birds, amphibians and snakes in sub-Saharan Africa. To summarize *a priori* the variability in the ensemble of 17 general circulation models, we introduce a consensus methodology that combines co-varying models. Thus, we quantify and map the relative contribution to uncertainty of seven bioclimatic envelope models, three multi-model climate projections and three emissions scenarios, and explore the resulting variability in species turnover estimates. We show that bioclimatic envelope models contribute most to variability, particularly in projected novel climatic conditions over Sahelian and southern Saharan Africa. To summarize agreements among projections from the bioclimatic envelope models we compare five consensus methodologies, which generally increase or retain projection accuracy and provide consistent estimates of species turnover. Variability from emissions scenarios increases towards late-century and affects southern regions of high species turnover centred in arid Namibia. Twofold differences in median species turnover across the study area emerge among alternative climate projections and emissions scenarios. Our ensemble of projections underscores the potential bias when using a single algorithm or climate projection for Africa, and provides a cautious first approximation of the potential exposure of sub-Saharan African vertebrates to climatic changes. The future use and further development of bioclimatic envelope modelling will hinge on the interpretation of results in the light of methodological as well as biological uncertainties. Here, we provide a framework to address methodological uncertainties and contextualize results.

## Introduction

Assessments of the potential effects of 21st century climatic changes on biodiversity commonly rely on bioclimatic envelope models (BEMs). Using correlations between climate and known species occurrences, BEMs estimate future shifts in suitable climate for species. Widespread use of BEMs has been accompanied by discussions of the biological (e.g. Pearson & Dawson, 2003; Araújo & Pearson, 2005; Sinclair *et al*., 2007) and methodological (e.g. Heikkinen *et al*., 2006; Beaumont *et al*., 2008) uncertainties that surround the outputs. BEMs rely on assumptions about the association between climate and species distributions, and their biological realism hinges on additional factors influencing species vulnerability to climatic changes, such as ecophysiological and micro-habitat preferences, phenotypic plasticity, evolutionary rates, dispersal ability (Chevin *et al*., 2010; Dawson *et al*., 2011; Hof *et al*., 2011), and biotic interactions (Araújo & Luoto, 2007; Suttle *et al*., 2007). In turn, BEM results are sensitive to the data and statistical functions that are used to describe the associations between species and climate. Alternative algorithms differ regarding the data used, variable selection, model parameterisation (Guisan & Zimmermann, 2000; Elith *et al*., 2006; Heikkinen *et al*., 2006), and techniques for extrapolation to novel conditions (Thuiller *et al*., 2004b; Pearson *et al*., 2006; Elith & Graham, 2009). BEMs are also sensitive to the greenhouse gas emissions scenarios and climate models used to simulate future climates (Beaumont *et al*., 2008).

Research is ongoing to develop more biologically realistic models (Keith *et al*., 2008; Anderson *et al*., 2009; Brook *et al*., 2009; Huntley *et al*., 2010; Kearney *et al*., 2010), but the breath of information and data required to appropriately parameterize them is large. Simpler approaches such as BEMs are thus likely to remain important tools for assessing potential impacts of climate change on biodiversity for a considerable time. In face of the increasing number of climate projections and statistical functions available, calls have been made to explicitly address the methodological uncertainties of BEMs so as to quantify the confidence that can be placed in forecasts (Thuiller *et al*., 2004a; Araújo *et al*., 2005b, 2006; Pearson *et al*., 2006; Diniz-Filho *et al*., 2009; Wiens *et al*., 2009; Buisson *et al*., 2010). The high level of certainty typically required for policy making can hardly be attained using correlative models—least of all under unknown future climates—leaving model users with the option of exploring the uncertainty in projections and weighing the risks associated with alternative actions (Wiens *et al*., 2009). One approach to address such uncertainties is to incorporate several assumptions and explore the resulting range of potential results (‘ensemble forecasting’, Araújo & New, 2007). Here, we provide the most extensive investigation to date of methodological uncertainties associated with ensemble forecasts of climate change impacts on sub-Saharan African vertebrate species.

The African continent is predicted to be one of the most vulnerable to 21st century climatic changes (Boko *et al*., 2007; Collier *et al*., 2008). Forecasts of warming above the global average (Christensen *et al*., 2007) are projected to affect African biodiversity and people's livelihoods (Velarde *et al*., 2005; Boko *et al*., 2007; Biggs *et al*., 2008). Yet, in comparison to well-researched regions, such as Europe or North America, Africa has received limited attention regarding the potential impacts of climate change on biodiversity (Lovett *et al*., 2005; Felton *et al*., 2009). Attribution of shifts in species distributions to climate change is difficult in Africa (Chown *et al*., 2010; Midgley & Thuiller, 2011) because changes in water availability—the main determinant of ecological responses (Hawkins *et al*., 2003)—are spatially complex and difficult to document (MacKellar *et al*., 2007). Increases in temperature, however, have been associated with the observed range extension of the Common Swift (Hockey & Midgley, 2009), and are likely to have more severe impacts for tropical species (Deutsch *et al*., 2008; Wright *et al*., 2009).

Previous studies using BEMs at a continental scale have projected substantial geographical shifts in suitable climate for African plants, birds and mammals by late-century (Table 1). Specifically, McClean *et al*. (2005) predicted losses of suitable climate for plant species in the Guineo-Congolian forests of western and central Africa, and gains in the surrounding uplands as well as the highlands of Namibia and the South African Drakensberg. Mammal species ranges around the equatorial zone in central Africa were projected to shift westward, with contractions in the Congo Basin, whereas mammals in southern Africa were projected to contract in the Kalahari region, and to shift eastward (Thuiller *et al*., 2006). For birds, forecasts revealed losses in southern and eastern Africa for breeding birds (Huntley *et al*., 2006) and trans-Saharan migrant passerines (Barbet-Massin *et al*., 2009), but relatively small changes for the former in equatorial and moist tropical forest habitats and even gains for the latter in the Sahel and Arabian Peninsula. Hole *et al*. (2009) also projected higher ensemble turnover for breeding birds in southern Africa, despite high persistence of suitable climate across the network of Important Bird Areas as a whole. The results presented in these studies are, however, contingent on the specific BEMs and future climate projections used. Only one study (Barbet-Massin *et al*., 2009) has fitted a range of different BEMs. Most of them also spanned a limited number of General Circulation Models (GCMs) and emissions scenarios, overlooking the variability among simulations of future climates which has been shown to be region-specific and relatively high for most of Africa south of the Equator (Hawkins & Sutton, 2009, 2011). Investigation of the level of uncertainty associated with the results was, thus, limited.

**Table 1:** Published continental- and sub-continental-scale studies using correlative models to assess the impacts of climate change on African biodiversity

Species data	Extent	Resolution	Modelling approach	Future scenarios	Reference
Distributional data for 5197 plant species (≥ 2 records)	Sub-Saharan	1^°^	• Box model, SGA and BGA	• 1 GCM: HadCM3	McClean *et al*., 2005;
				• 1 SRES: B1	
	Africa			• 3 time periods: 2025, 2055 and 2085	
Distributional data for bird species breeding in Africa	Sub-Saharan	1^°^	• Locally weighted regression	• 3 GCMs: HadCM3, GFDL_R30, ECHAM4/OPYC3	Huntley *et al*., 2006;
	Africa			• 1 SRES: B2	
				• 1 time period: 2070–99	
Extent of occurrence data for 277 mammal species	Africa	10′	• GAM	• 1 GCM: HadCM3	Thuiller *et al*., 2006;
				• 2 SRES: A2 and B2	
				• 2 time periods: 2050 and 2080	
Distributional data for 1608 bird species (≥ 5 records)	Sub-Saharan	1^°^	• CRS and GAM	• 3 GCMs: HadCM3, ECHAM4, GFDL-R30	Hole *et al*., 2009;
	Africa			• 1 SRES: B2a	
				• 3 time periods: 2025, 2055 and 2085	
Distributional data for 64 bird species (≥ 6 records)	Sub-Saharan Africa	0.5^°^	• GLM, GAM, CTA, ANN, MDA, MARS, GBM, RF, MaxEnt	• 5 GCMs: BCM2, ECHAM5, HadCM3, MIROHIC3_2-HI and MK3)	Barbet-Massin *et al*., 2009
			• Consensus projection	• 3 SRES: A1B, B1 and A2	
				• 3 time periods: 2030, 2065 and 2100	

ANN, artificial neural networks; GCM, general circulation model; AUC, area under the receiving operator curve; BGA, Bayes-based genetic algorithm; CRS, species-climate response surfaces; CTA, classification tree analysis; GAM, generalized additive model; GBM, generalized boosting model; GLM, generalized linear model; MARS, multivariate adaptive regression splines; MaxEnt, Maximum Entropy; MDA, mixture discriminant analysis; RF, random forests; SGA, simple genetic algorithm; SRES, special report emission scenario.

In this article, we use seven BEM techniques to describe the bioclimatic envelopes of 284 amphibian, 310 snake, 623 mammal and 1506 bird species in sub-Saharan Africa. To assess the impacts of climatic changes on the modelled species, we project their envelopes to mid- and late-century climates. We use an ensemble of 17 GCMs, under the B1, A1B and A2 emissions scenarios from the Intergovernmental Panel on Climate Change (IPCC). As large ensembles of projections are difficult to interpret, consensus methodologies can be used to average across ensembles. Multi-model climate projections are widely used in climatology, but there is still debate on the best consensus methodologies to combine models (Tebaldi & Knutti, 2007; Knutti *et al*., 2010). To retain information about the full variability in our ensemble of GCMs, we introduce a methodology that averages co-varying GCMs based on their similarity in both magnitude and spatial pattern. We thus reduce the ensemble of GCMs to three multi-model projections, and obtain, for each of the 2723 species modelled, 126 projections of bioclimatic envelopes (seven BEM techniques, three climate projections, three emissions scenarios, and two time periods), or 343 098 projections overall. To facilitate interpretation of this ensemble, we also summarize agreements among projections from the seven BEMs. Consensus methodologies have been used in previous climate change ecology work in Europe (Thuiller, 2004; Araújo *et al*., 2006, 2011; Buisson & Grenouillet, 2009; Thuiller *et al*., 2011), the Americas (Diniz-Filho *et al*., 2009, 2010; Lawler *et al*., 2009; Marini *et al*., 2009, 2010; Roura-Pascual *et al*., 2009), Asia (Ogawa-Onishi *et al*., 2010), Australia (Crossman & Bass, 2008) and Africa (Barbet-Massin *et al*., 2009; Coetzee *et al*., 2009), and have generally yielded higher accuracy than single-models. Yet, there is still debate on the best methodologies for combining BEM projections and only a few comparisons have been published (Araújo *et al*., 2005b; Araújo & New, 2007; Marmion *et al*., 2009). Here, we compare five methodologies, including the methodology introduced to combine GCMs.

Our projections provide insights into the potential exposure of sub-Saharan African vertebrates to 21st century climatic changes. We explicitly address the variability in forecasts of species temporal turnover from alternative climate projections and BEMs, or combinations thereof. More specifically, we investigate: a) the relative contribution of different sources of uncertainty in forecasts of species turnover; b) the predictive accuracy of forecasts from alternative BEM consensus methodologies; and c) the variation in forecasts with alternative BEM consensus methodologies and climate projections.

## Data and methods

### Species and climate data

The study region covered continental sub-Saharan Africa, south of 20°N. Species occurrence data for amphibian (Hansen *et al*., 2007a), snake (Rasmussen *et al*., 2007), mammal (Galster *et al*., 2007) and terrestrial bird species (Hansen *et al*., 2007b) in sub-Saharan Africa were used from the 1° resolution (≍111 km x 111 km at the Equator) databases held at the Zoological Museum within the University of Copenhagen in Denmark. This is the most comprehensive biodiversity dataset for Africa, compiled from multiple sources and continuously improved over 15 years (Burgess *et al*., 1998; Brooks *et al*., 2001). Data were available for 741 amphibians, 477 snakes, 1085 mammals and 1789 birds. Because of statistical difficulties with modelling species with limited numbers of occurrence records (Stockwell & Peterson, 2002; Wisz *et al*., 2008), we excluded species with fewer than 15 records over the study area. Our threshold may introduce uncertainty in the analysis, yet the effect of sample size on model accuracy is a question that requires further study. More conservative thresholds for sample size have been suggested (e.g. Harrell *et al*., 1984), but some algorithms have been shown to achieve 90% of their maximum accuracy with ten records (Stockwell & Peterson, 2002). In total 2723 species were modelled, accounting for 67% of the available data (284 amphibians, 310 snakes, 623 mammals and 1506 birds, with median range sizes of 71, 90, 94 and 162 grid cells respectively).

Baseline climate data averaged for the 1961-90 period were obtained from the Climatic Research Unit (New *et al*., 2002) at a resolution of 10′ (≍ 18.6 km × 18.6 km at the Equator). Monthly precipitation and mean temperature values were used to compute 21 variables that are commonly useful in bioclimatic modelling studies (see Appendix S1 in Supporting Information). We applied principal components analysis (PCA) to identify sets of uncorrelated variables that represent major climatic gradients over the study area. From the first axis, we selected the variable with the highest loading, i.e., annual precipitation. An additional variable was selected from the first axis with high loading, but opposite sign (correlation −0.45): temperature of the warmest month. The variable with the highest loading on the second axis, i.e., temperature of the coldest month, was the third variable selected. The two-first axes explained 74.3% of the variation (Appendix S1). Together, precipitation and temperature influence water availability, which controls biological activity in the tropics and sub-tropics (Hawkins *et al*., 2003). Both precipitation- and temperature-based variables are important determinants of the distributions of bird species (Huntley *et al*., 2006) and a variety of other species in Africa (Chown, 2010 and references therein). The temperature-based variables selected in our study further reflect the important effect of seasonal temperatures on species’ distributions (Huntley *et al*., 2006).

Future climate projections were derived from 17 GCMs downscaled to 10′ resolution (Tabor & Williams, 2010; see Appendix S2). All downscaled GCMs were from the World Climate Research Programme's Coupled Model Intercomparison Project phase 3 multi-model dataset projections (Meehl *et al*., 2007) and de-biassed using the change-factor technique and observational data from the CRU. The datasets comprise monthly mean temperature and precipitation projections for the 2041-60 and 2081-00 time intervals. Simulations from the 17 GCMs were used for three illustrative greenhouse gas emissions scenarios from the IPCC's Special Report Emissions Scenarios (Nakicenovic *et al*., 2000). We used high- to low-end scenarios (A2, A1B and B1) that reflect different assumptions about demographic, socio-economic and technological development on greenhouse gas emissions. For each GCM projection and scenario, we computed the three selected variables over the study area. To match the species data resolution, both baseline and future climate datasets were re-sampled in ArcGIS (ESRI, 2006), using bilinear interpolation, to the 1° grid over sub-Saharan Africa. Data processing and statistical analyses were performed using R (R Development Core Team, 2010) version 2.11.1.

### Combining ensembles of climate projections

We first summarized the general tendencies among the 17 selected GCMs. In climatology, multi-model ensemble averages have often been shown to improve the outcome of climate simulations (Phillips & Gleckler, 2006; Tebaldi & Knutti, 2007; Gleckler *et al*., 2008; Reichler & Kim, 2008; Pierce *et al*., 2009; Knutti *et al*., 2010; but see Fordham *et al*., *in press*). However, averaging ensembles can result in the loss of higher-order variability reflected in extreme projections (Beaumont *et al*., 2008). To avoid this limitation, we used a hybrid consensus approach (Araújo *et al*., 2006) – hereafter referred to as ‘central cluster’ – that groups co-varying projections before averaging them. When there is great variation in projections –as it is often the case with precipitation – this approach also avoids averaging projections that are very different or even contradictory, by placing them in different groups.

To combine the GCMs, we used three steps in R (R Development Core Team, 2010; see Appendix S3 for the R scripts). First, as a basis for identifying co-varying projections under each emissions scenario, we assessed similarities among GCM simulations for each variable projected in the late-century, when inter-simulation spread becomes larger (Stott & Kettleborough, 2002; Meehl *et al*., 2007; Hawkins & Sutton, 2009). Similarities were assessed separately for each variable because the performance of climate models varies for different variables (Lambert & Boer, 2001; Gleckler *et al*., 2008). We used model performance metrics to characterize the agreement between individual simulations for each variable and the multi-model median ensemble for the same variable. These metrics were spatially aggregated point-wise measures of regional deviations (Duan & Phillips, 2010). The spatial pattern Pearson correlation (R) reflects spatial agreement between individual simulations of a given variable and the median simulation of that variable. The signed standardized anomaly (D), in turn, measures signed differences in magnitude between individual simulations and the median simulation of a given variable, standardized using the standard deviation of all simulations. D thus reflects whether a simulation tends to under- or over-estimate a given variable in relation to the median of all simulations of that variable, and by how much. D values close to zero and R values close to 1 indicate high similarity between a given model and the multi-model median.

Second, the ensemble of 17 GCMs was partitioned into groups of co-varying projections according to the 2081-00 D and R statistics obtained. We used *k-means*, a clustering technique that assigns data points to the closest pre-defined centre. These centres were the median points of single linkage hierarchical clusters based on the Euclidean distance matrix (Venables & Ripley, 2003). The significance of the differences between clusters was tested with Anosim, a nonparametric test of analysis of similarity (Clarke & Warwick, 1994). Anosim was applied to the dissimilarity matrix of D and R values to test whether the distances between clusters were greater than the distances within clusters. The initial number of clusters to extract was selected so as to minimize inter-cluster distances in the hierarchical trees, and was increased when needed until the Anosim test was statistically significant. For each emissions scenario we obtained a set of clusters, each with a number of co-varying late-century climate models. The same clusters were applied to the baseline and 2041-60 time periods.

Third, we generated summaries of the patterns of central tendency in each cluster extracted. For each cluster of co-varying GCMs obtained for different emissions scenarios and time slices, un-weighted median consensus forecasts were computed on each variable. There are contrasting views on the use of weights to perform climate ensemble averages. They have been shown not to systematically change the results in some cases (e.g. Pierce *et al*., 2009) and to improve them in other cases (e.g. Min & Hense, 2006). Which model performance metrics to use as weights also remains an issue of debate (Tebaldi & Knutti, 2007; Gleckler *et al*., 2008). As the optimal performance weights for future projections are unlikely the same as for baseline climate (Tebaldi & Knutti, 2007), we opted for un-weighted averages. In summary, for each emissions scenario and time period combination we obtained a set of clusters of GCMs, each with the median simulation of each variable computed across GCMs. For each set, the cluster with the average D closest to zero and the highest average R captured the maximum consensus among projections, corresponding to the ‘central cluster’, whereas clusters departing from the multi-model median ensemble captured extreme projections.

### Bioclimatic envelope modelling

Models were fitted at 1° resolution using seven presence-absence BEM techniques in BIOMOD (Thuiller *et al*., 2009), a computing platform for ensemble forecasting that operates in R environment (R Development Core Team, 2010). The techniques included three regression methods (generalized linear models (GLM), generalized additive models (GAM) and multivariate adaptive regression splines (MARS)), three machine-learning methods (artificial neural networks (ANN), Breiman and Cutler's random forest for classification and regression (RF), and generalized boosting models (GBM)), and one classification method (flexible discriminant analysis, FDA). Due to differences in quality, species occurrence data were treated differently across taxa. Estimated range maps for mammals and birds, based on numerous records of species across multiple countries, lend themselves to be treated as presence-absence data. For most amphibians and snakes, however, the data comprise confirmed specimen locality records from museum collections and thus were considered to be closer to presence-only data. For the latter taxa, pseudo-absences were randomly generated to allow fitting models that assume the data to be in the form of presences and absences. The process of generating random pseudo-absences in BIOMOD weighs them to achieve a prevalence of 0.5. There is a debate on how to select pseudo-absences, and the choice of selection method is dependent on the purpose of the study (Chefaoui & Lobo, 2008). Yet, random selection has been shown to result in higher predictive power than strategies that select pseudo-absences from low-suitability regions (Wisz & Guisan, 2009).

For each species, the seven models were built using a calibration subset of 75% of the sites selected at random and evaluated with the remaining 25% of the sites. This data-splitting procedure was repeated five times. Projections of the probability of occurrence of species in each site were converted to binary format (presence/absence) using a threshold maximizing the True Skill Statistic (TSS, Allouche *et al*., 2006). The models were evaluated based on median omission and commission errors and TSS on the cross-validated data. The calibrated models were used to generate projections of species’ bioclimatic envelopes under each GCM cluster and emissions scenario for 2041-60 and 2081-00. The projections were based on the final runs of the models using 100% of the data, as data partitions have been shown to add significant uncertainty to forecasts (Araújo *et al*., 2005b, 2009). As we were interested in measuring changes in climatic suitability for species rather than interpreting model projections as estimates of the changes in observed species distributions, we adopted an ‘unlimited dispersal’ scenario, whereby species are assumed to be able to track shifting suitable climate over the entire study area. To complement these projections, the areas where higher proportions of species are projected to retain climatic suitability over time (corresponding to a ‘no dispersal’ assumption) were also mapped.

### Combining ensembles of BEM forecasts

For each taxon, we explored the agreement among projections from the seven BEM techniques using five consensus methodologies. The first three methodologies provide a synthetic measurement of the central tendency in the frequency distribution of the projections obtained from all BEMs. Implemented within the BIOMOD package, the ‘ensemble mean’ computes the un-weighted mean (e.g. Buisson & Grenouillet, 2009; Diniz-Filho *et al*., 2010), the ‘ensemble weighted mean’ (e.g. Marmion *et al*., 2009) uses the TSS values as weights, and the ‘ensemble median’ calculates the second quartile of the frequency distribution of forecasts from all models (e.g. Araújo *et al*., 2005b; Lawler *et al*., 2009). The fourth and fifth methodologies, in turn, preselect projections that best summarize consensus among them.

With the fourth method – ‘central model’ – we selected the model summarizing the highest amount of variation among projections. For each species, PCAs were performed on the projected probabilities within the BIOMOD package, and the ‘central model’ corresponded to the one with the highest PCA loading in the first (consensus) axis (e.g. Thuiller *et al*., 2005; Algar *et al*., 2009). In the fifth method – ‘central cluster’ – we investigated patterns of central tendency among groups of co-varying projections (e.g. Araújo *et al*., 2005b, 2006). Following the approach used to combine the ensemble of GCMs, we clustered the BEMs based on the similarities between single-BEM probabilistic projections and the multi-model median probabilistic projection. We used the same measures of D and R, computed for each species. Unlike the other four consensus methodologies, the grouping was performed for the set of all species in one taxon rather than individually for each species. The same procedure of clustering and Anosim testing we used for GCM projections was followed. The corresponding binary projections for the BEMs in each cluster were combined using a majority vote criterion, whereby a species was considered present in grid points where more than half of the BEMs in the cluster predicted presence. The ‘central cluster’ was the closest to the multi-model projection. Both the PCA and cluster analysis were applied to the end-of-century scenario, when divergence is expected to be highest, and the same model(s) used to derive consensus forecasts in the baseline and 2041-60 time periods.

The BEM consensus projections were built using 100% of the data for the same reasons cited above for single-BEM projections. To evaluate the consensus projections, we applied the same five consensus methodologies to the five evaluation datasets (25% of the data) and computed the median omission error, commission error and TSS of all cross-validations and, in the case of amphibians and snakes, all pseudo-absence runs. To assess the level of consensus among BEMs, we performed PCAs for each species on the probabilistic projections, both for all seven BEMs and for the ‘central cluster’ BEMs only. The proportion of variance explained by the first principal component axis provided a measure of consensus among projections (Thuiller, 2004; Araújo *et al*., 2006; Grenouillet *et al*., 2011).

### Mapping shifts in climatic suitability and associated uncertainties

For each emissions scenario and GCM cluster combination, and for the five BEM consensus methodologies applied, baseline and future species richness and the number of contracting and expanding species were computed. Spatial patterns of change were investigated using measures of species temporal turnover per grid cell (Peterson *et al*., 2002). The ‘species turnover rate’ refers to local dissimilarities between baseline and future sets of species for which a given area is projected to be climatically suitable, and thus incorporates both losses and gains of climate space. In addition, *in situ* persistence of climatic suitability for species was investigated.

To evaluate and map the relative contributions of emissions scenarios, future climates and BEMs to the overall uncertainty in forecasts, we performed analyses of variance (anova) in R (R Development Core Team, 2010). Following Diniz-Filho *et al*. (2009), we performed a three-way anova without replication for each grid cell, using the turnover rate as the response variable and the emissions scenarios, future climate projections and BEM consensus methodologies as factors. An anova using single-BEMs, climate projections and emissions scenarios as factors, in turn, provided indications on the relative contribution of individual BEMs to uncertainty in turnover projections (before combining the ensembles). To explore BEM uncertainties associated with model extrapolation, we mapped the areas projected to experience future climates beyond the range of climate values used to fit the models for each of the three variables. For each GCM cluster and scenario combination, the sum of these areas corresponded to ‘non-analogue’ areas, where projections become statistically less reliable (Heikkinen *et al*., 2006; Williams *et al*., 2007; Fitzpatrick & Hargrove, 2009).

## Results

### Relative contribution of different sources of uncertainty in forecasts of species turnover

**Fig. 1:**
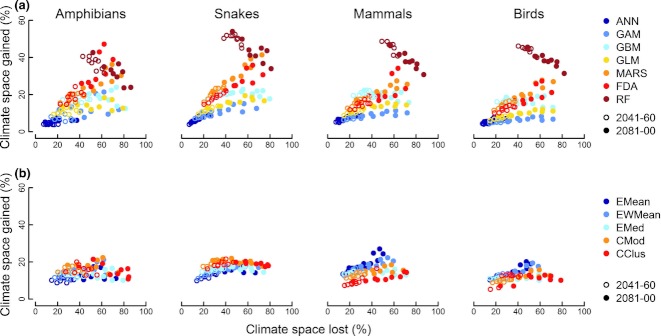
Proportion of suitable climate projected to be lost and gained by species for the seven bioclimatic envelope models (BEM) (a) and the five BEM consensus projections (b) under alternative climate projections. Values are median percentages of grid cells lost or gained for all species of amphibians (*n* = 284), snakes (*n* = 310), mammals (*n* = 623) and birds (*n* = 1506) in mid- (open circles) and late-century (solid circles). For a given time period, each circle corresponds to one of the nine combinations of three emissions scenarios and three general circulation model (GCM) clusters. The BEMs are Artificial Neural Networks (ANN), Generalized Additive Models (GAM), Generalized Boosting Model (GBM), Generalized Linear Models (GLM), Multivariate Adaptive Regression Splines (MARS), Flexible Discriminant Analysis (FDA) and Random Forests (RF), and the BEM consensus are ensemble mean (EMean), ensemble weighted mean (EWMean), ensemble median (EMed), central model (CMod) and central cluster (CClus).

Uncertainty in species turnover forecasts was mainly caused by the variability among BEMs. In the point-wise anova using BEMs, GCM clusters and emissions scenarios as factors, the median proportions of the total sum of squares across the study area attributed to BEMs reached between 76% for mammals and 82% for snakes by mid-century (Appendix S4). The relative contribution of BEMs to overall uncertainty decreased by late-century (to between 61% for mammals and 69% for snakes) due to increased divergence among emissions scenarios. Variability across BEMs was strongly affected by RF projections that displayed higher losses and gains of suitable climate across climates and emissions scenarios, departing from the general trend across models (Fig. 1a; see below).

**Fig. 2:**
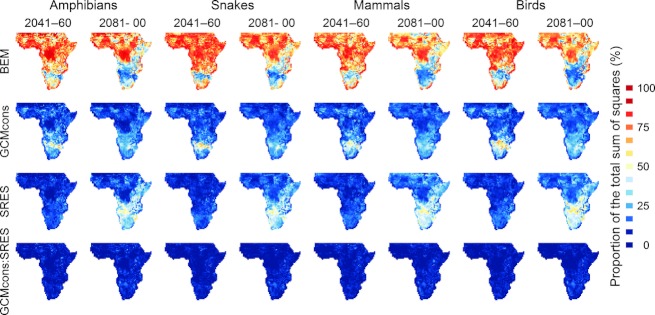
Spatial variation of the relative contribution of future climate projections and bioclimatic envelope models to the variability in species turnover forecasts for amphibian, snake, mammal and bird species. Values shown correspond to the proportion of the total sum of squares accounted for by the bioclimatic envelope models (BEM), general circulation model clusters (GCMcons), emissions scenarios (SRES), and one interaction factor (GCMcons:SRES) in the three-way analysis of variance (anova) performed for each grid cell over the study area (*N* = 1851) on turnover forecasts for each taxon. Data are shown for mid- and late-century.

**Fig. 3:**
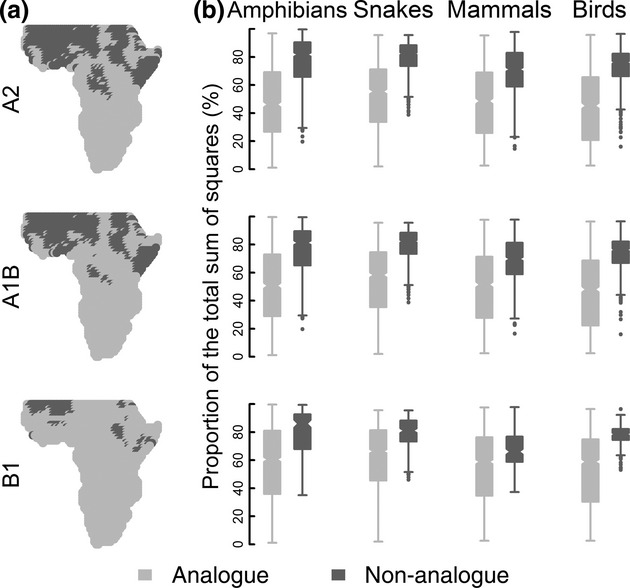
Comparison of uncertainties arising from bioclimatic envelope models between analogue and nonanalogue climate grid cells, for amphibians, snakes, mammals and birds. The maps (a) show the distribution over the study area (*N* = 1851) of nonanalogue climates (dark grey) for scenarios A2 (*N* = 738 non-analogue grid cells), A1B (*N* = 632) and B1 (*N* = 374). The graphs (b) show the frequency of the distributions of the proportions of the total sum of squares accounted for by bioclimatic envelope models in the point-wise three-way analysis of variance (anova) performed for each taxon using species turnover projections as response variable. The difference is shown between analogue (light grey) and nonanalogue (dark grey) climate grid cells over the study area (Kolmogorov–Smirnov tests *P*-value < 0.001 across taxa). Data refer to the ‘maximum consensus’ general circulation model cluster (cluster 2) under the three emissions scenarios.

Disagreement among BEM forecasts in late-century was mainly concentrated in the northern half of the study area, particularly in Sahelian and southern Saharan Africa (Fig. 2). These areas were predicted to experience mean temperatures of the warmest and coldest months above the calibration range (Fig. 3a; Appendix S5), forcing the models to extrapolate beyond known relationships. Late-century nonanalogue climates covered up to 50% of the study area for the most severe climate projection. A comparison of grid points with analogue and nonanalogue climates (Fig. 3b) revealed significant differences in the proportion of the total sum of squares attributed to BEMs for all time periods and future climates (Kolmogorov–Smirnov tests *P*-value < 0.001). The northern regions of high BEM uncertainty mostly corresponded to areas with nonanalogue climates.

### Predictive accuracy of forecasts from alternative BEM consensus methodologies

**Fig. 4:**
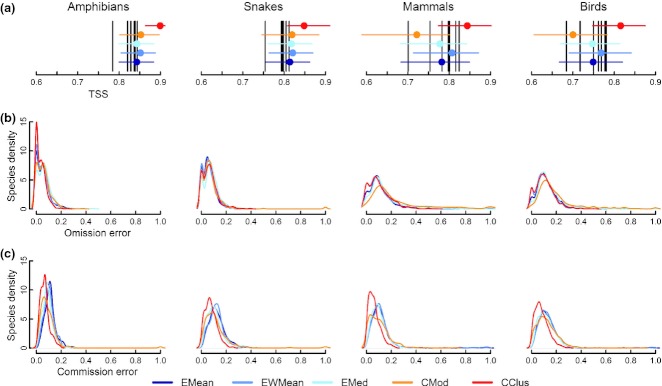
Comparison of average True Skill Statistics (TSS) between bioclimatic envelope models (BEM) and BEM consensus projections (a), and density functions of omission error (b) and commission error (c) of BEM consensus projections for all species in each taxon. Omission and commission error and TSS values refer to the median of the validation datasets (and pseudo-absence runs in the case of amphibians and snakes). The TSS plots (a) show the median (full circles) and the upper and lower quartiles (the extremes of the horizontal lines) of the TSS values of all species for each BEM consensus projection, as well as the median TSS values of all species for each BEM projection (black vertical lines). The consensus projections are the ensemble mean (EMean), ensemble weighted mean (EWMean), ensemble median (EMed), central model (CMod) and central cluster (CClus). The CMod and CClus projections shown were built using late-century projections from the GCM cluster 2 under A1B. The individual models are Artificial Neural Networks (ANN), Generalized Additive Models (GAM), Generalized Boosting Model (GBM), Generalized Linear Models (GLM), Multivariate Adaptive Regression Splines (MARS), Flexible Discriminant Analysis (FDA) and Random Forests (RF).

Using the median TSS across all species as an evaluation criterion, we found that consensus projections outperformed or equalled all or six of the single-BEMs for amphibians and snakes, and between one and seven single-BEMs for mammals and birds (Fig. 4a). The ‘central cluster’ methodology provided the most robust projections for all taxa, surpassing all single-BEMs. This methodology resulted in four clusters for each taxon, with the ‘central cluster’ combining high-accuracy models (GAM and GLM for mammals, GAM, GBM, GLM and RF for birds, and GBM and GLM for amphibians and snakes for most GCM cluster and scenario combinations; Anosim *P*-value < 0.05, see Appendix S6 for results on 100% of the data). The ‘central cluster’ projections were also the most consensual, raising the median levels of consensus across all species for the four taxa, although to different degrees depending on the number and spread of projections in the cluster (Appendix S7). The ‘central model’ methodology, in turn, yielded high accuracy projections for amphibians and snakes, but the lowest accuracy of all consensus projections for mammals and birds. Whereas for a large fraction of amphibian and snake species the ‘central model’ corresponded to high-accuracy models (GBM and ANN), for mammal and bird species the selection covered a wider range of models (see Appendix S8 for results on 100% of the data) with varying levels of accuracy, yielding lower median TSS across species.

High accuracy of consensus projections was linked to both low omission and commission errors (Fig. 4b and c). The ‘central cluster’ projections displayed lower numbers of known absence points incorrectly predicted (Fig. 4c; for each taxon, distribution of commission errors across species significantly different from the remaining consensus projections according to Wilcoxon signed rank tests, see Appendix S9). For birds in particular, the models combined in the ‘central cluster’ projections included RF, which incorporate the notion of ensemble forecasting (Araújo & New, 2007) and can yield highly accurate projections (Prasad *et al*., 2006). The extreme discrepancy in commission error of RF models from the other projections, however, suggests that these models may have over-fitted the training data (see Jimenez-Valverde *et al*., 2008). For amphibians and snakes, the measurements of commission error may have been affected by random pseudo-absences placed in climatically suitable areas (Anderson *et al*., 2003, Peterson *et al*., 2006).

### Variation in forecasts with alternative BEM consensus methodologies and climate projections

**Fig. 5:**
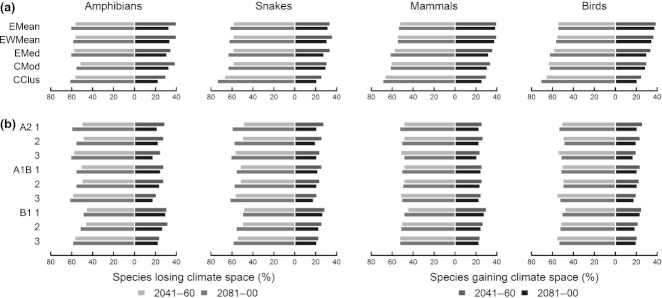
Percentage of species predicted to lose or gain suitable climate consistently across the three emissions scenarios (a) and across the five BEM consensus methodologies (b). The percentages of species of amphibians (*n* = 284), snakes (*n* = 310), mammals (*n* = 623) and birds (*n* = 1506) that are consistently projected to contract (lighter tones, left side of graphs) or expand (darker tones, right side of graphs) their bioclimatic envelopes for the ‘maximum consensus’ general circulation model cluster across all emissions scenarios (A2, A1B and B1) are shown for each consensus methodology (a). The percentages of species in each taxon that are consistently projected to contract or expand across all BEM consensus projections (EMean, ensemble mean; EWMean, ensemble weighted mean; EMed, ensemble median; CMod, central model; CClus, central cluster) are shown for each climate projection (b). Data are shown for mid- and late-century.

When the BEMs were combined and the relative sources of uncertainty re-assessed in a point-wise anova, differences emerged across taxa. For amphibians and snakes, mid-century turnover forecasts varied most with the BEM consensus methodologies (median proportions of the total sum of squares across the study area of 37% and 43% respectively, decreasing to 24% and 29% by late-century; see Table 2). For these taxa, ‘central cluster’ median estimates of late-century species turnover over the study area were up to 1.6 times higher than those produced by the most conservative BEM consensus methodologies across all climate projections (Appendix S10). By contrast, only up to 1.2-fold differences resulted for mammal and bird species. Geographically, the five BEM consensus projections displayed similar patterns of species turnover for each taxon, with larger variations for amphibians and snakes between the ‘central cluster’ and the remaining consensus projections (Appendix S11). For each taxon, there were consistent trends among consensus methodologies of median losses and gains for all species across climate projections (Fig. 1b). Consistent across all taxa was a trend towards more species contracting their bioclimatic envelopes than expanding. Depending on the BEM consensus projection, between 54% and 74% of the species in each taxon were consistently estimated to lose suitable climate across emissions scenarios by late-century (Fig. 5a).

**Table 2:** Relative contribution of general circulation models and bioclimatic envelope model consensus methodologies to overall uncertainty in species turnover projections for amphibian, snake, mammal and bird species. The values are proportions of the total sum of squares from the three-way analysis of variance (anova) performed for each grid cell over the study area (*N* = 1851) to evaluate the relative contributions of the bioclimatic envelope model consensus methodologies (BEMcons), the general circulation model clusters (GCMcons), the emissions scenarios (SRES) and interactions among these factors, to the variability in turnover forecasts for each taxon. Values correspond to the median and (in brackets) the lower and upper quartiles of the proportions of the total sum of squares attributed to each factor over the study area, and are shown for both mid- and late-century

	Amphibians	Snakes	Mammals	Birds
2041–60				
BEMcons	**36.5** (20.4–60.5)	**42.8** (24.8–60.6)	**24.7** (11.8–44.1)	**20.6** (11.4–34.9)
GCMcons	**24.4** (9.2–40.7)	**25.7** (11.6–41.3)	**34.1** (19.0–50.4)	**40.2** (24.5–54.7)
SRES	**9.8** (4.4–16.3)	**10.7** (6.0–16.7)	**15.2** (9.5–21.7)	**18.2** (11.7–25.7)
BEMcons:GCMcons	**3.4** (1.7–6.4)	**3.1** (1.8–5.2)	**3.2** (1.8–5.9)	**2.0** (1.1–3.3)
BEMcons:SRES	**1.9** (0.9–3.8)	**1.9** (1.1–3.4)	**2.3** (1.3–4.1)	**2.0** (0.9–4.7)
GCMcons:SRES	**4.2** (2–7.6.0)	**3.9** (2.1–6.8)	**4.3** (2.2–7.5)	**4.5** (2.4–8.1)
BEMcons:GCMcons:SRES	**4.3** (2.2–8.5)	**3.7** (2.1–5.9)	**4.3** (2.5–7.5)	**2.4** (1.3–4.4)
2081–00				
BEMcons	**23.6** (7.6–48.8)	**28.8** (11.3–47)	**12.7** (3.6–29.3)	**12.4** (3.3–24.7)
GCMcons	**19.2** (8.9–27.7)	**19.8** (11.7–27.2)	**24.7** (17.6–31.1)	**28.0** (21.3–34.6)
SRES	**28.5** (13.3–48.7)	**32.1** (19.6–48.8)	**44.2** (31.4–57.1)	**45.8** (32.3–57.3)
BEMcons:GCMcons	**1.9** (0.9–3.7)	**1.5** (0.7–3.2)	**1.4** (0.6–2.8)	**1.0** (0.4–1.8)
BEMcons:SRES	**1.8** (0.9–3.7)	**1.7** (0.9–3.3)	**1.9** (0.8–3.7)	**1.4** (0.6–2.7)
GCMcons:SRES	**4.3** (2.2–8.2)	**3.8** (2.0–6.9)	**3.6** (2.0–6.1)	**4.0** (2.0–6.8)
BEMcons:GCMcons:SRES	**3.4** (1.5–8.5)	**2.6** (1.5–5.9)	**2.4** (1.1–4.7)	**1.2** (0.7–2.3)

In contrast, alternative climate projections had the largest impact on mid-century projections for mammals and birds, with the GCM clusters explaining 34% and 40% respectively of the total sum of squares in the anova (Table 2). Towards the end of the century, the spread across emissions scenarios increased, becoming the major source of uncertainty for all taxa. For each scenario, the three clusters of GCMs obtained (Anosim statistics 0.72 (A1B) and 0.81 (B1 and A2), *P* = 0.001) reflected a warming gradient. The maximum consensus ‘central cluster’ (cluster 2) reflected intermediate levels of warming. The other clusters captured low- (cluster 1) and high-end (cluster 3) temperature variability across climate models. For the temperature of the warmest month, for example, median values of late-century anomalies projected over the study area varied between 1.8 °C (lower and upper quartiles 1.6–2.0%) for the low-end cluster 1 under B1 and 5.1 °C (4.6–5.7 °C) for the high-end cluster 3 under A2 (similar patterns emerged for the temperature of the coldest month; see Appendix S12). Trends across the GCM clusters were less clear for precipitation forecasts, and sometimes showing contrasting directions of change (Appendix S13), but cluster 3 was consistently the driest for all scenarios. Following the warming gradient, median late-century turnover rates across the study area almost doubled from cluster 1 under B1 to cluster 3 under A2 (Appendix S10).

**Fig. 6:**
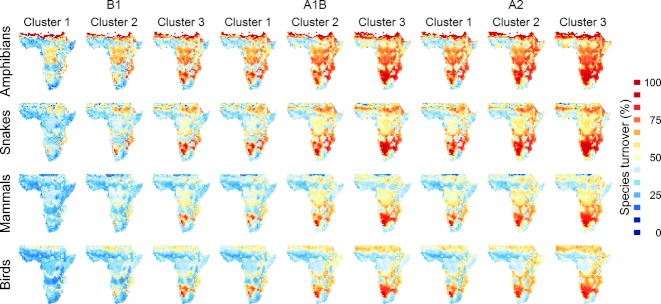
Projected late-century species turnover rate (%) for amphibian, snake, mammal and bird species under the alternative emissions scenarios (A2, A1B and B1) and general circulation model clusters (Clusters 1, 2 and 3). Data refer to the ensemble median of all bioclimatic envelope model projections.

**Fig. 7:**
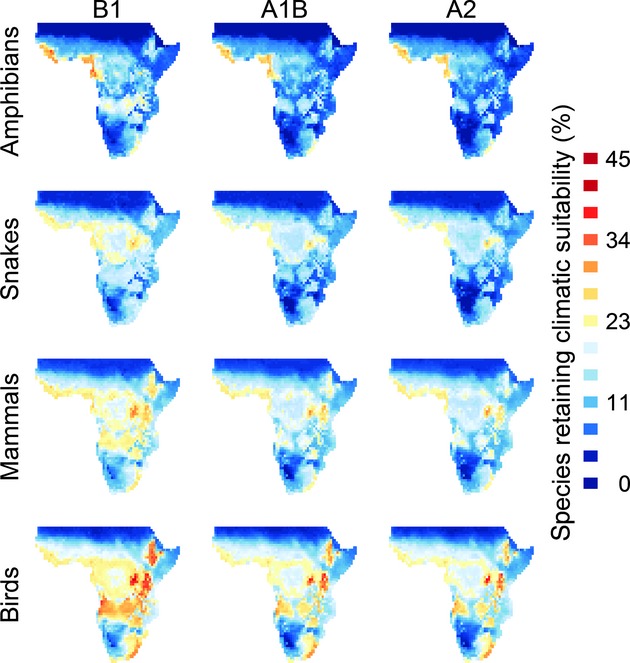
Percentage of species predicted to retain climatic suitability under each emissions scenario. The proportion of the total numbers of species of amphibians (*n* = 284), snakes (*n* = 310), mammals (*n* = 623) and birds (*n* = 1506) that are projected to retain climatic suitability in each location are shown for the median ensemble of all bioclimatic envelope models and for the ‘maximum consensus’ general circulation model cluster under the A2, A1B and B1 emissions scenarios.

For all taxa, a southern area centred in the arid regions of Namibia emerged with high turnover rates by late-century (Fig. 6). However, the geographical extent of this high-turnover effect varied from isolated patches of the Kalahari in Namibia and Botswana and of southern Mozambique for cluster 1 under B1, to most of inland Namibia, Botswana and southern Mozambique for cluster 3 under A2. A comparison of the areas projected to remain climatically suitable for species over time revealed more striking differences across taxa (Fig. 7). For amphibians, it was the West African forests that were projected to remain climatically suitable for the largest proportion of species, irrespective of climate projection. A significant proportion of snake species were also projected to persist in this area, but under the B1 scenario also in the Albertine Rift mountain forests and extending around the Congo Basin. In comparison, larger climatically stable areas were projected for mammal and bird species, particularly under B1, with the highest proportions of species persisting in the Ethiopian highland mountain, Albertine Rift, East Africa montane and Eastern Arc forests, as well as the Angolan scarp and Miombo woodlands and the Drakensberg and eastern coast of South Africa for bird species. Projections for cluster 1 under B1 showed the lowest proportions of species of all taxa consistently predicted to contract across all BEM consensus projections (Fig. 5b).

## Discussion

We explored the spread in estimates of climatically suitable areas for African vertebrates using alternative climate projections and emissions scenarios, as well as BEMs and combinations thereof. Our aim was to address the methodological uncertainty associated with the results. Other methodological factors that are not accounted for in our analysis are likely to add further uncertainty to the projections. They include gaps and biases in species occurrence data (Hortal *et al*., 2008), the selection of predictors (Synes & Osborne, 2011), the temporal (Roubicek *et al*., 2010) and spatial resolution (Kriticos & Leriche, 2010) of baseline climate data, and the thresholds used to convert probabilistic to binary projections (Araújo *et al*., 2005b; Nenzén & Araújo, 2011). Yet, the uncertainty arising from future climate projections and from BEMs, which differ in how they extrapolate to nonanalogue climates, has particular relevance in the climate change context.

### Sources of uncertainty

Although comparisons of sources of uncertainty in forecasts depend on the amount of variability captured by each source (Diniz-Filho *et al*., 2009), our assessment spanned a wide variability for the three sources considered: seven classes of BEM that perform differently when projected to the future (Thuiller *et al*., 2004a; Araújo *et al*., 2006; Pearson *et al*., 2006), three emissions scenarios that span a good portion of the range of the six IPCC scenarios (Manning *et al*., 2010), and 17 of the 23 IPCC fourth Assessment Report GCMs. In line with previous studies (Araújo *et al*., 2005b; Pearson *et al*., 2006; Diniz-Filho *et al*., 2009, 2010; Nenzén & Araújo, 2011), mid-century projections were most affected by the choice of BEM (Fig. 2). The relative amount of variation introduced by BEMs, however, decreases over time (Buisson *et al*., 2010 and this study), as divergence among emissions scenarios increases (Stott & Kettleborough, 2002; Hawkins & Sutton, 2009). By late-century, twofold differences emerged in species turnover projections with alternative GCM clusters and emissions scenarios.

Differences among projections from alternative climate projections were especially important in southern Africa (Fig. 2), where some of the greatest changes in species turnover were projected (Fig. 6). In contrast, variability from BEMs was especially high in the northern half of the study area (Fig. 2), coinciding with projected nonanalogue climates (Fig. 3). The problem of model extrapolation into 21st century novel climates has been overlooked in most continental-scale studies in Africa and elsewhere, although there have been efforts to quantify it at different geographical scales, for example in Europe (Saetersdal *et al*., 1998; Thuiller *et al*., 2004b; Araújo *et al*., 2011), North America (Roberts & Hamann, in press) and Australia (Fitzpatrick & Hargrove, 2009; Elith *et al*., 2010). Projected novel climate conditions are unevenly distributed worldwide (Williams *et al*., 2007). In Africa, forecasts of severe warming increase the risk of nonanalogue climates, particularly under high-end emissions scenarios (Williams *et al*., 2007; Appendix S5).

Solutions to the problem of extrapolation into nonanalogue climates include classifying such areas as species absences (Austin & Meyers, 1996; Thuiller *et al*., 2004b), excluding them from the analysis (Saetersdal *et al*., 1998), or using a larger calibration area before projecting to the region of interest (Pearson *et al*., 2006). Yet, such solutions may lead to misleading results when the bioclimatic envelope of the species has not been fully captured by the calibration data or when the species is not in equilibrium with climate (Svenning & Skov, 2004; Araújo & Pearson, 2005). With long time horizons, novel climates are expected to become more widespread (Williams *et al*., 2007), and the problem of extrapolation may persist even when using larger calibration areas, and all the more when the species response curves are high or increasing where truncated (Anderson & Raza, 2010; Webber *et al*., 2011). The ideal solution of using ecological or physiological knowledge of the species to classify nonanalogue climate areas as either presences or absences (Elith *et al*., 2010) is not feasible for most species where these data are lacking. The approach used in our study may thus reflect the current best practice: mapping nonanalogue climates to identify where uncertainties are likely to be higher (Platts *et al*., 2008; Fitzpatrick & Hargrove, 2009; Elith *et al*., 2010; Araújo *et al*., 2011; Roberts & Hamann, in press) and using a range of BEMs that make different assumptions about the responses of species in those areas (Pearson *et al*., 2006).

### Role of consensus approaches

Although multi-model averages of GCMs have rarely been used by ecologists (but see Beaumont *et al*., 2011; Roberts & Hamann, in press), their value for global change studies has been recognized (Beaumont *et al*., 2008; Fordham *et al*., in press). Our ensemble of GCMs reflects availability, and may thus not sample the full range of uncertainty or guarantee independence of models (Meehl *et al*., 2007; Tebaldi & Knutti, 2007; Knutti *et al*., 2008), but the large number of models included is expected to minimize potential biases by model choice (Knutti *et al*., 2010). Our approach to combine groups of co-varying GCMs enabled us to retain information about the full variability of projections, including those that are extreme, and minimize the effect of combining often divergent precipitation projections. In face of the wealth of climate simulations available, this approach might prove increasingly useful in ecological modelling studies.

How alternative methodologies to combine BEMs affect forecasts of species bioclimatic envelopes has rarely been investigated (but see Araújo *et al*., 2005b, 2006; Marmion *et al*., 2009). Measurements of model accuracy in the baseline context do not necessarily provide an indication of the models’ ability to transfer into future conditions (Araújo *et al*., 2005a). However, in the absence of independent data for evaluation of the models, they can be used as benchmark for verification of the consistency of alternative consensus methodologies. Using a variety of methodologies, we found that consensus projections displayed greater consistency in accuracy for amphibians and snakes. Whereas the three consensus methodologies averaging the full ensemble of BEM projections were generally consistent for all taxa, for mammals and birds they diverged from those methodologies combining only preselected models (Fig. 4a). This distinction across taxa resulted from the greater spread of projection accuracy from the seven BEMs for mammals and birds. For all taxa, however, the ‘central cluster’ methodology stood out as the most accurate projection. In the case of amphibians and snakes, accurate and constrained baseline ‘central cluster’ projections led to higher species turnover rates than the remaining consensus methodologies (Appendix S11).

### Interpreting BEM outputs

The climatically suitable areas identified in this study are areas where the exposure of species to climatic changes can potentially allow the persistence of vertebrates through time. Assessing the potential response of species to these changes would necessitate mechanistic information about their sensitivity and adaptive capacity. Correlative studies have used such information as a source of data to infer absence (Elith *et al*., 2010) or presence points (Hijmans & Graham, 2006), as model predictors (Rödder *et al*., 2009), or as complementary information (Morin & Thuiller, 2009), but similar applications to large datasets like ours are limited by data availability. Information on species’ dispersal capacity, for example, would determine whether projected new suitable climate space is accessible to species. Given the known variation in dispersal capacity among the four taxa, effective range shifts would diverge more across taxa than the projected gains of suitable climate (Fig. 1). Mapping areas that remain climatically suitable for most species through time reflects potential persistence of species *in situ* (Fig. 7), whereas integrating dispersal rate estimates in BEMs (Midgley *et al*., 2006) or combining BEM and migration model projections (Iverson *et al*., 2004) would indicate potential range shifts. Biotic factors such as habitat structure further limit the response of vertebrates. African habitats are controlled by the interaction among climate, atmospheric CO_2_ and disturbances like fire (Scheiter & Higgins, 2009). In the case of grass-dominated savannas, the low levels of CO_2_ that triggered their development during the last glacial are clearly being surpassed, with expected changes in tree cover (Bond *et al*., 2003; Kgope *et al*., 2010) and associated fauna (Sirami *et al*., 2009). The use in BEMs of vegetation predictors derived from mechanistically based dynamic vegetation models (Triviño *et al*., in press) could capture these effects and, to some extent, reduce uncertainties arising in nonanalogue situations.

Exposure of species to climatic changes was measured in our study by three climatic variables. The realism of our models thus depends on the relationship between species distributions and these variables. This relationship is unlikely to be constant through time, not only for its complexity but also for the changing correlations among climatic variables (Morin & Lechowicz, 2008). Decoupling of patterns of covariance between predictor and proximal variables may undermine the value of BEMs when extrapolating in time (Jackson *et al*., 2009; Elith *et al*., 2010). Our variables also discount climatic variability nested across temporal and spatial scales. Multi-decadal climatic means fail to capture fluctuations and rapid transitions between climate states (Jackson *et al*., 2009). Projected changes in mean precipitation in Southern Africa, for example, differ between seasons and do not always parallel those in extreme precipitation (Shongwe *et al*., 2009). However, it is the interplay between temporal variability and species survival thresholds that determines the effects on species (Jackson *et al*., 2009). By the same token, the coarse spatial resolution used here overlooks microclimates provided by topographical or vegetation features (e.g. Shoo *et al*., 2011).

BEM outputs need to be interpreted in the light of the methodological uncertainty explored and the biological limitations discussed above. If carefully implemented, BEMs can, therefore, provide a first-order, parsimonious assessment of the changes in the distribution of suitable climate for species (Thuiller *et al*., 2008; Jackson *et al*., 2009; Chevin *et al*., 2010; Huntley *et al*., 2010). A new generation of models that couple correlative with mechanistic approaches (Keith *et al*., 2008; Anderson *et al*., 2009; Brook *et al*., 2009; Huntley *et al*., 2010) is required to allow predictions of population persistence that have direct relevance to local or regional conservation (Jackson *et al*., 2009; Chevin *et al*., 2010). Yet our ensemble forecasting implementation of BEMs provides general insights into the potential exposure of sub-Saharan African vertebrates to climate change at the continental scale, as well as a critical background for those seeking to interpret these results and use them as the basis for decision-making at this large scale.

## Supporting Information

**Appendix S1.** Principal Components Analysis to select predictor variables.

**Appendix S2.** General Circulation Models used in the study.

**Appendix S3.** R scripts used to build the ‘central cluster’ consensus projections.

**Appendix S4.** Sources of uncertainty in the ensemble of forecasts from individual BEMs.

**Appendix S5.** Non-analogue climate maps.

**Appendix S6.** Single-BEMs selected for the ‘central clusters’ and Anosim results.

**Appendix S7.** Level of consensus among all BEMs and ‘central cluster’ BEMs.

**Appendix S8.** Single-BEMs selected as ‘central model’ across species.

**Appendix S9.** Statistical differences in the distributions of TSS and omission and commission error among the five BEM consensus projections.

**Appendix S10.** Frequency distribution of species turnover for alternative GCM clusters, emissions scenarios and BEM consensus methodologies.

**Appendix S11.** Late-century species turnover for alternative BEM consensus methodologies.

**Appendix S12.** Frequency distribution of climate anomalies over the study area.

**Appendix S13.** Climate anomaly maps for the three variables.
