# Supplementary material for: Exploring consensus in 21st century projections of climatically suitable areas for African vertebrates
Source: Glob Chang Biol. 2011 Dec 30;18(4):1253–69. doi: 10.1111/j.1365-2486.2011.02605.x (PMC3597255; doi:10.1111/j.1365-2486.2011.02605.x)
Supplement: Supplementary file 1 [file gcb0018-1253-SD1.pdf]

Appendix S1: Principal Components Analysis to select predictor variables

21 predictor variables were computed using baseline monthly precipitation and temperature data for 1961-60 from the Climatic Research Unit (CRU, New *et al.*, 2002). A principal components analysis (PCA) was performed on the values of these variables over the study area (N=1,851) in order to select a smaller set of variables and minimise co-linearity. The loadings for the first three principal components (PC1 to 3) are shown. The last three rows show the standard deviation, proportion of variance explained and cumulative variance explained by each PC.

**Variables**	**PC1**	**PC2**	**PC 3**
mean temperature of the coldest month	0.022	**0.365**	0.016
mean temperature of the warmest month	**0.277**	0.194	-0.023
annual mean temperature	0.196	0.305	-0.028
annual sum of precipitation	**-0.295**	0.161	0.095
annual mean precipitation	-0.295	0.161	0.095
precipitation of the driest month	-0.194	0.080	-0.558
precipitation of the wettest month	-0.231	0.172	0.348
precipitation of the coldest month	-0.143	0.171	0.036
precipitation of the warmest month	-0.250	0.023	-0.208
mean temperature of the driest month	0.050	0.345	-0.027
mean temperature of the wettest month	0.268	0.161	-0.162
mean temperature of the coldest quarter	0.042	0.364	0.022
mean temperature of the warmest quarter	0.272	0.203	-0.038
precipitation of the driest quarter	-0.209	0.097	-0.538
precipitation of the wettest quarter	-0.246	0.158	0.343
precipitation of the coldest quarter	-0.160	0.190	0.050
precipitation of the warmest quarter	-0.270	0.020	-0.108
mean temperature of the driest quarter	0.068	0.355	-0.010
mean temperature of the wettest quarter	0.272	0.165	-0.147
temperature seasonality	0.159	-0.275	-0.057
precipitation seasonality	0.282	-0.010	0.167
**Standard Deviation**	2.936	2.642	1.301
**Proportion of Variance Explained**	0.410	0.332	0.081
**Cumulative Proportion of Variance**	0.410	**0.743**	0.823
