# Supplementary figures and images for: Exploring consensus in 21st century projections of climatically suitable areas for African vertebrates
Source: Glob Chang Biol. 2011 Dec 30;18(4):1253–69. doi: 10.1111/j.1365-2486.2011.02605.x (PMC3597255; doi:10.1111/j.1365-2486.2011.02605.x)

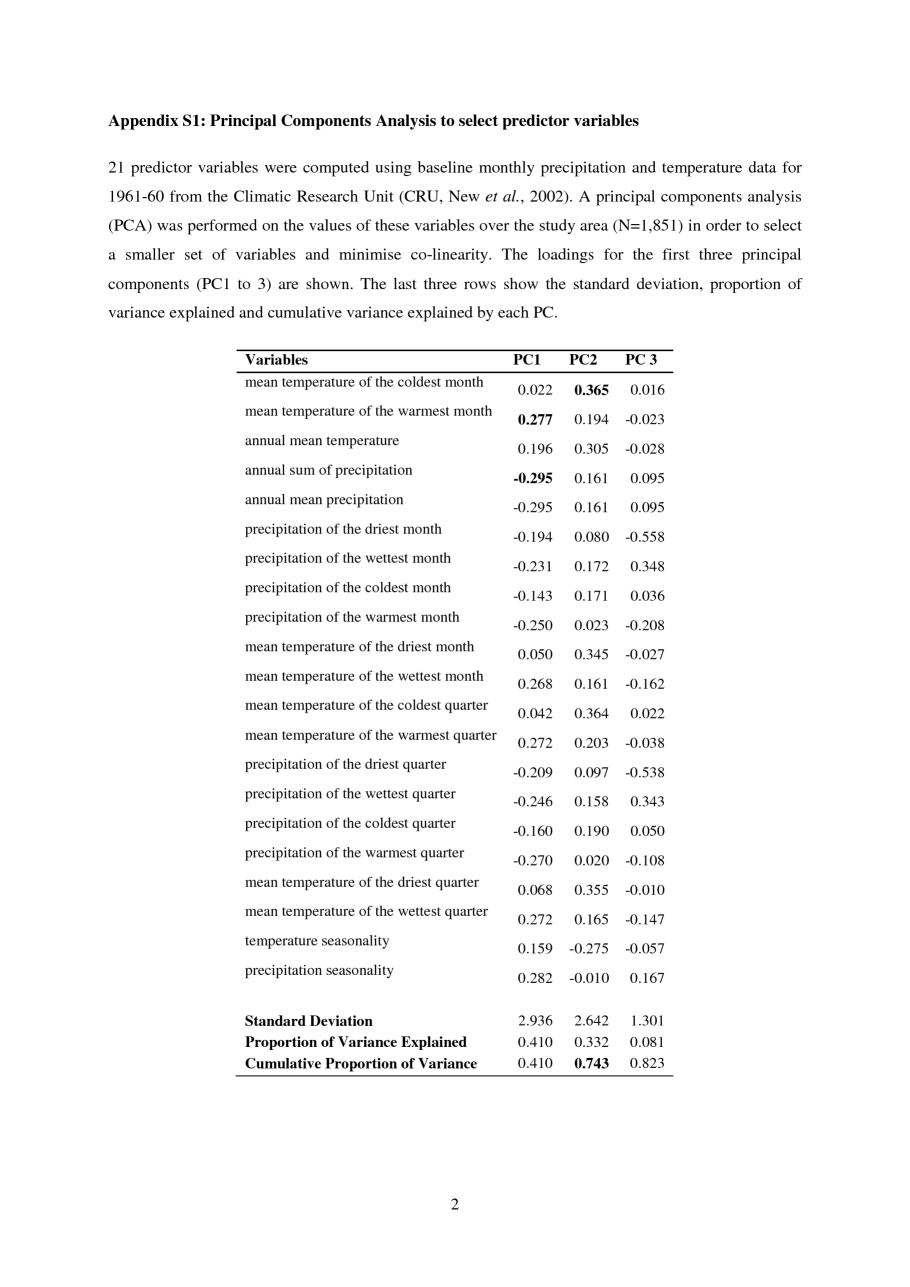

Supplement: Supplementary file 2 [file gcb0018-1253-SD14.png]

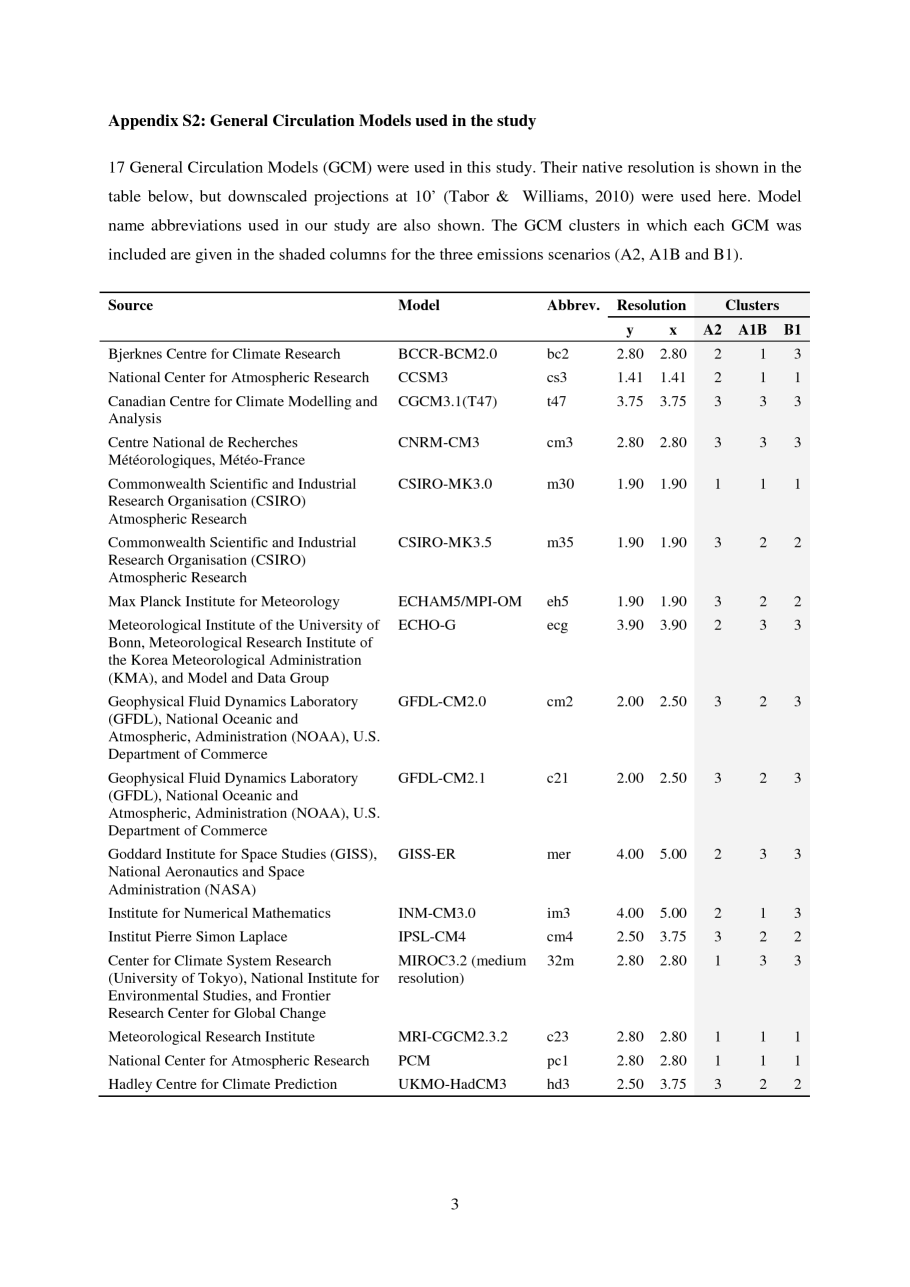

Supplement: Supplementary file 4 [file gcb0018-1253-SD15.png]

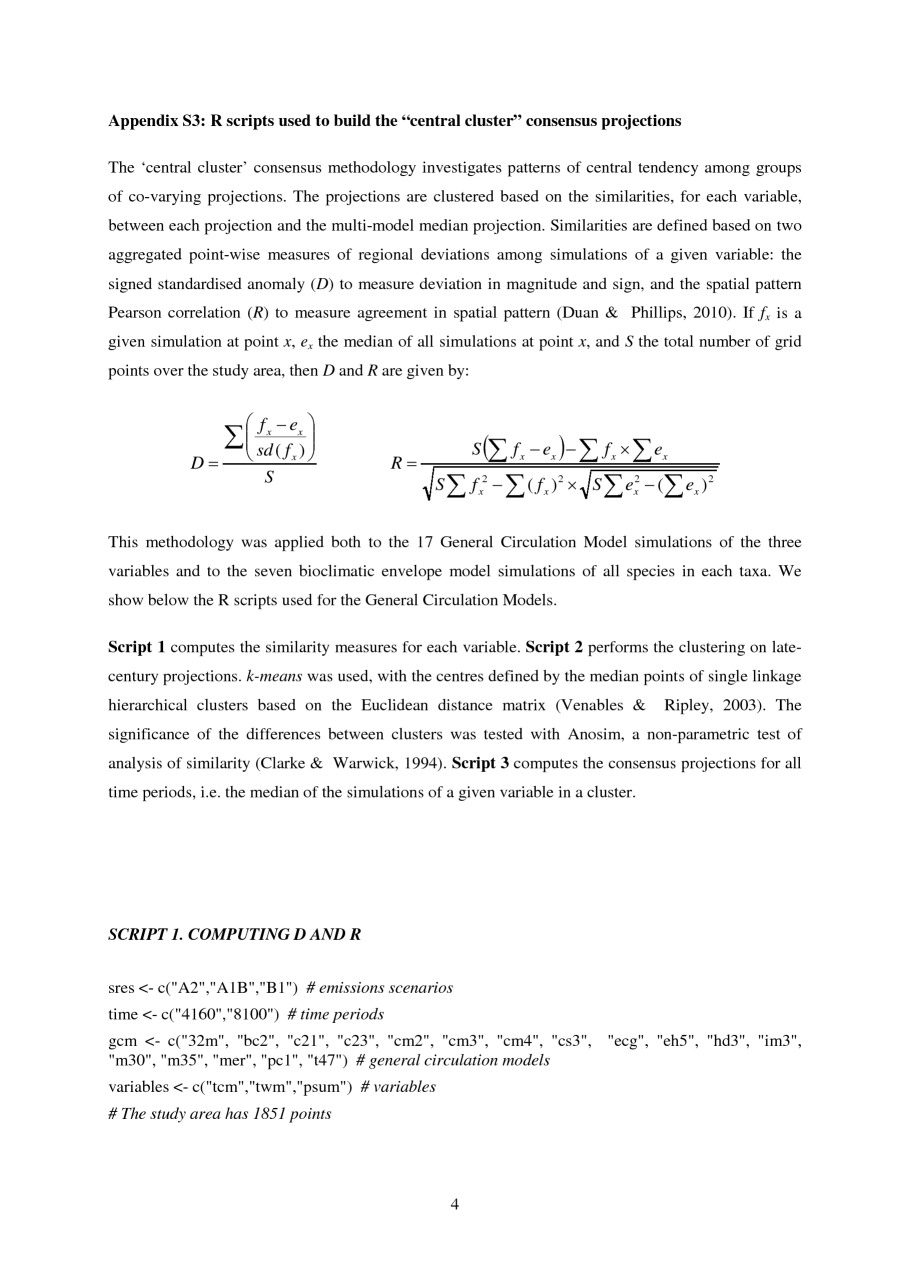

Supplement: Supplementary file 6 [file gcb0018-1253-SD16.png]

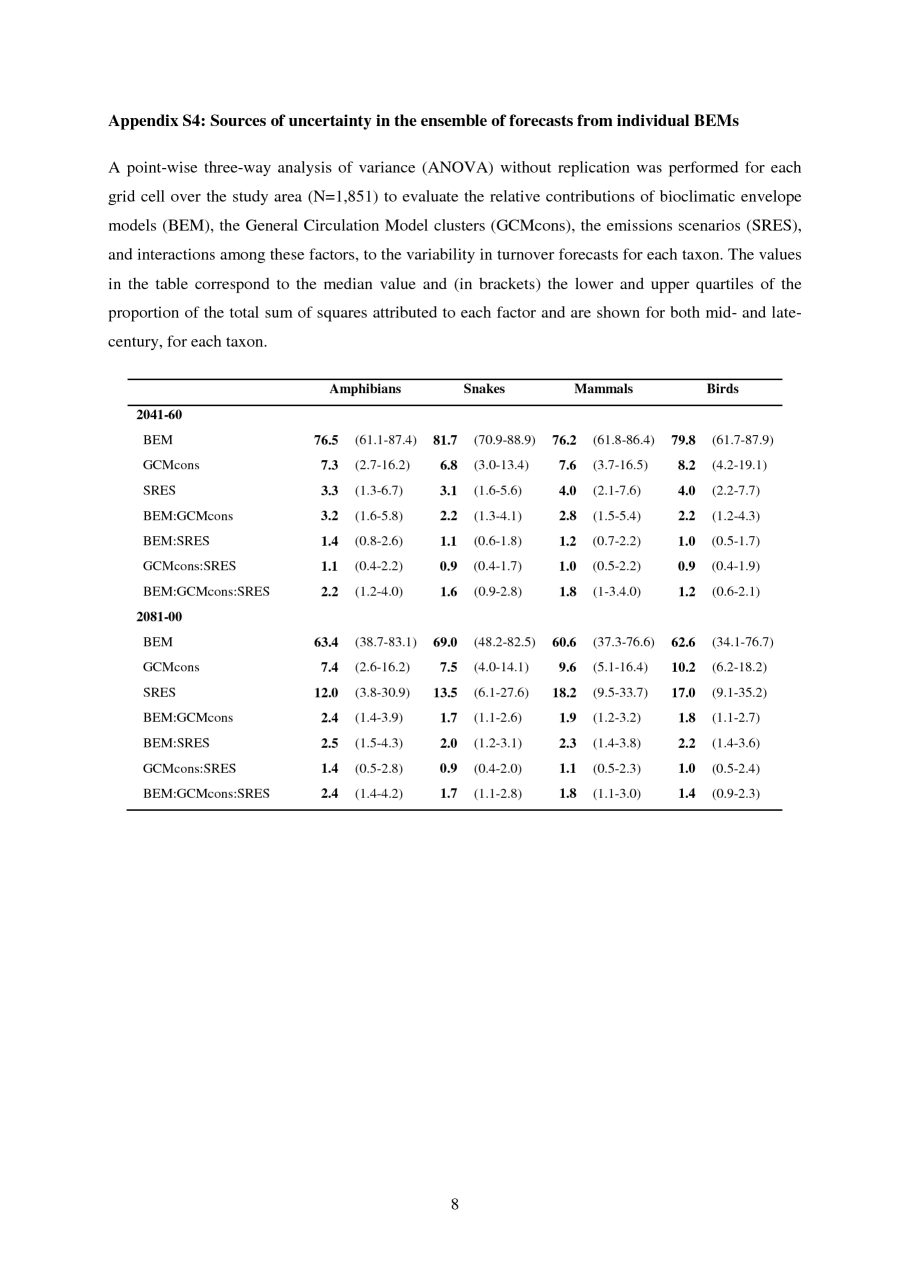

Supplement: Supplementary file 8 [file gcb0018-1253-SD17.png]

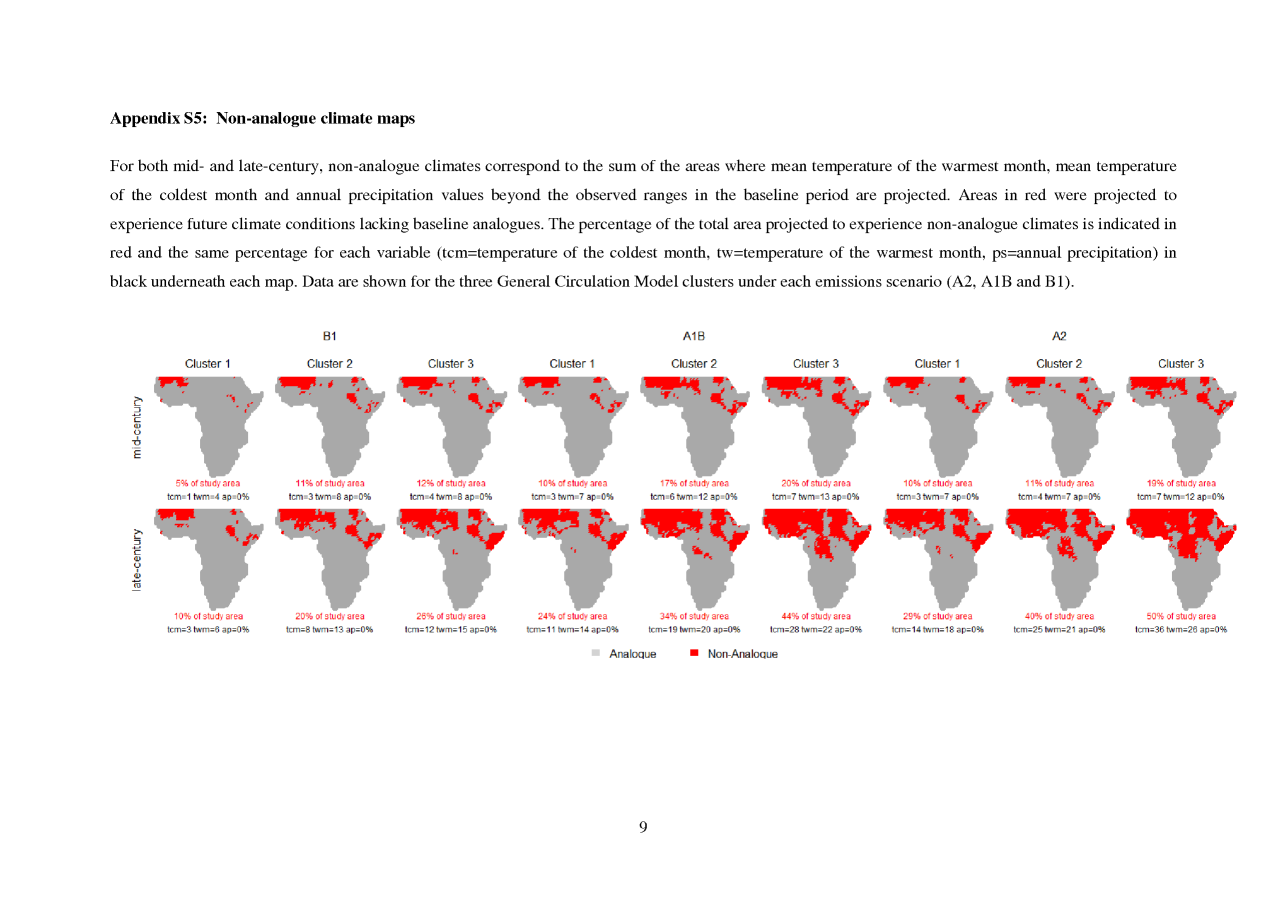

Supplement: Supplementary file 10 [file gcb0018-1253-SD18.png]

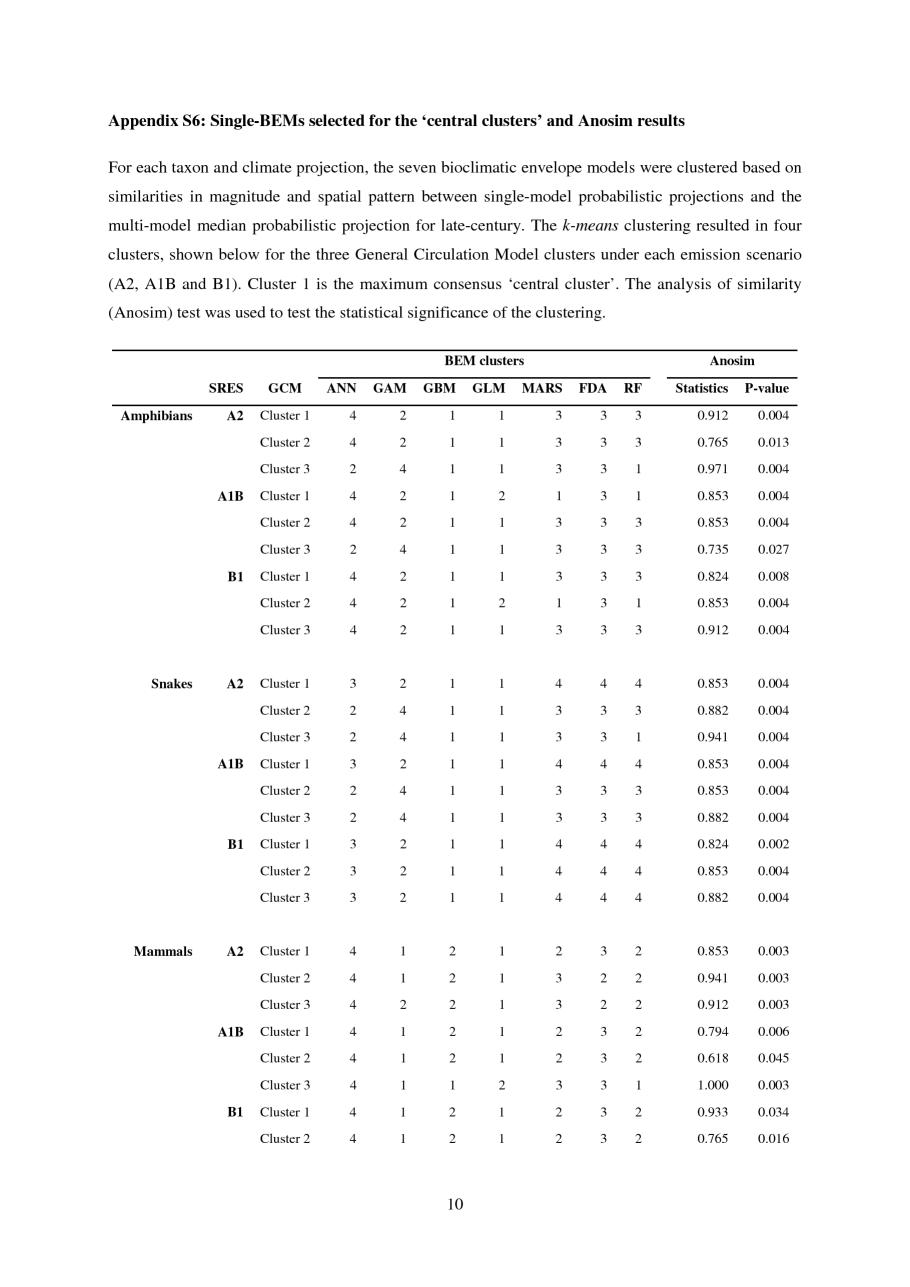

Supplement: Supplementary file 12 [file gcb0018-1253-SD19.png]

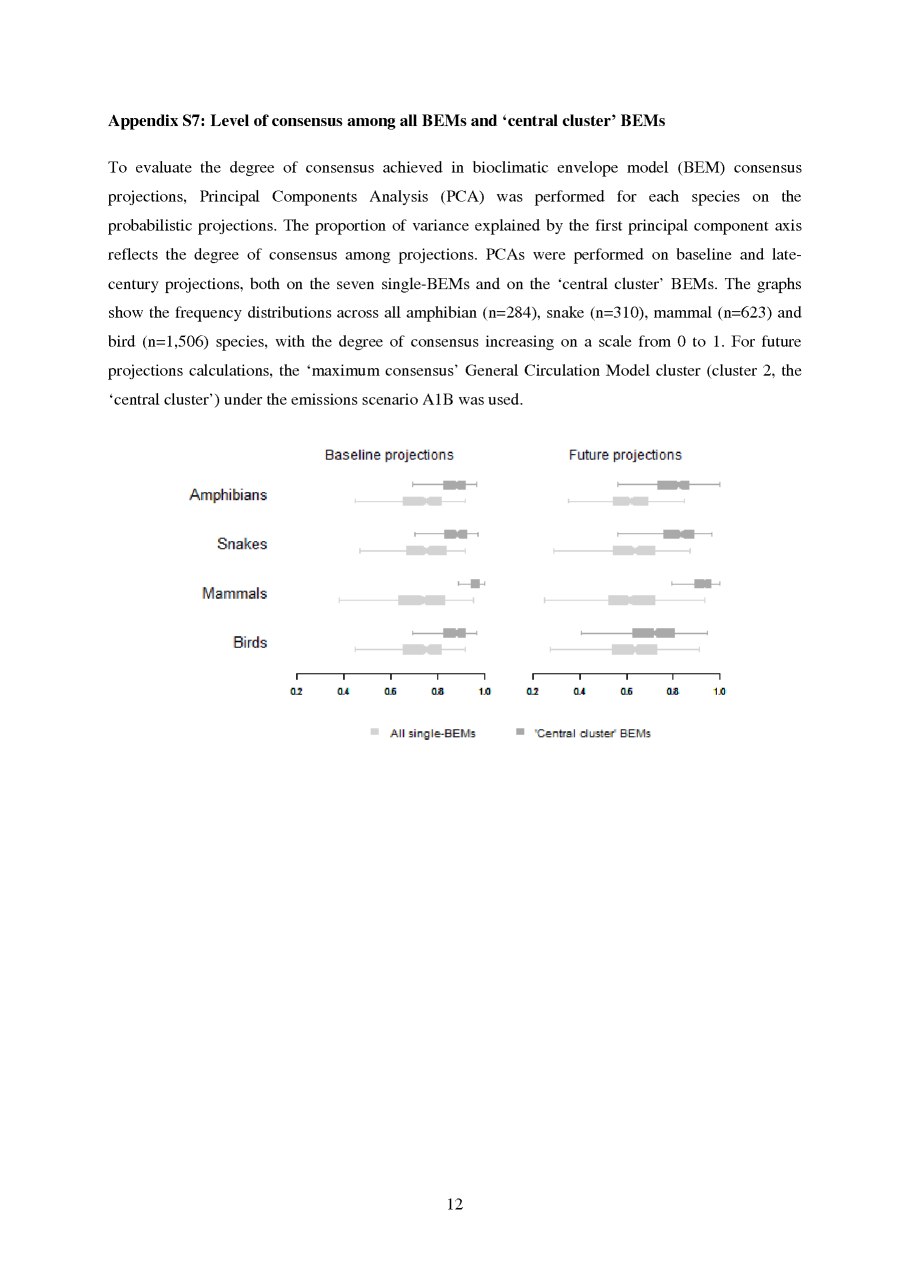

Supplement: Supplementary file 14 [file gcb0018-1253-SD20.png]

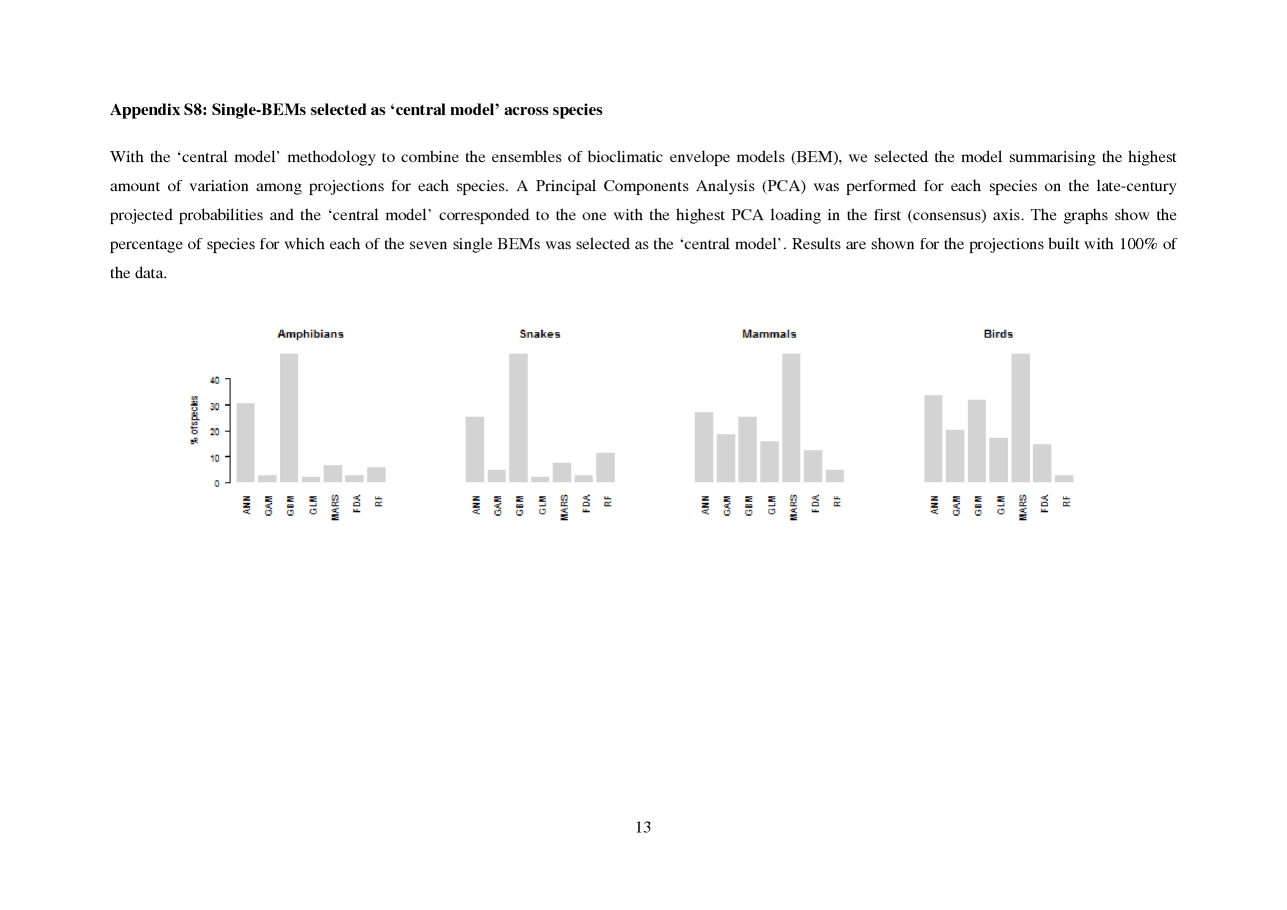

Supplement: Supplementary file 16 [file gcb0018-1253-SD21.png]

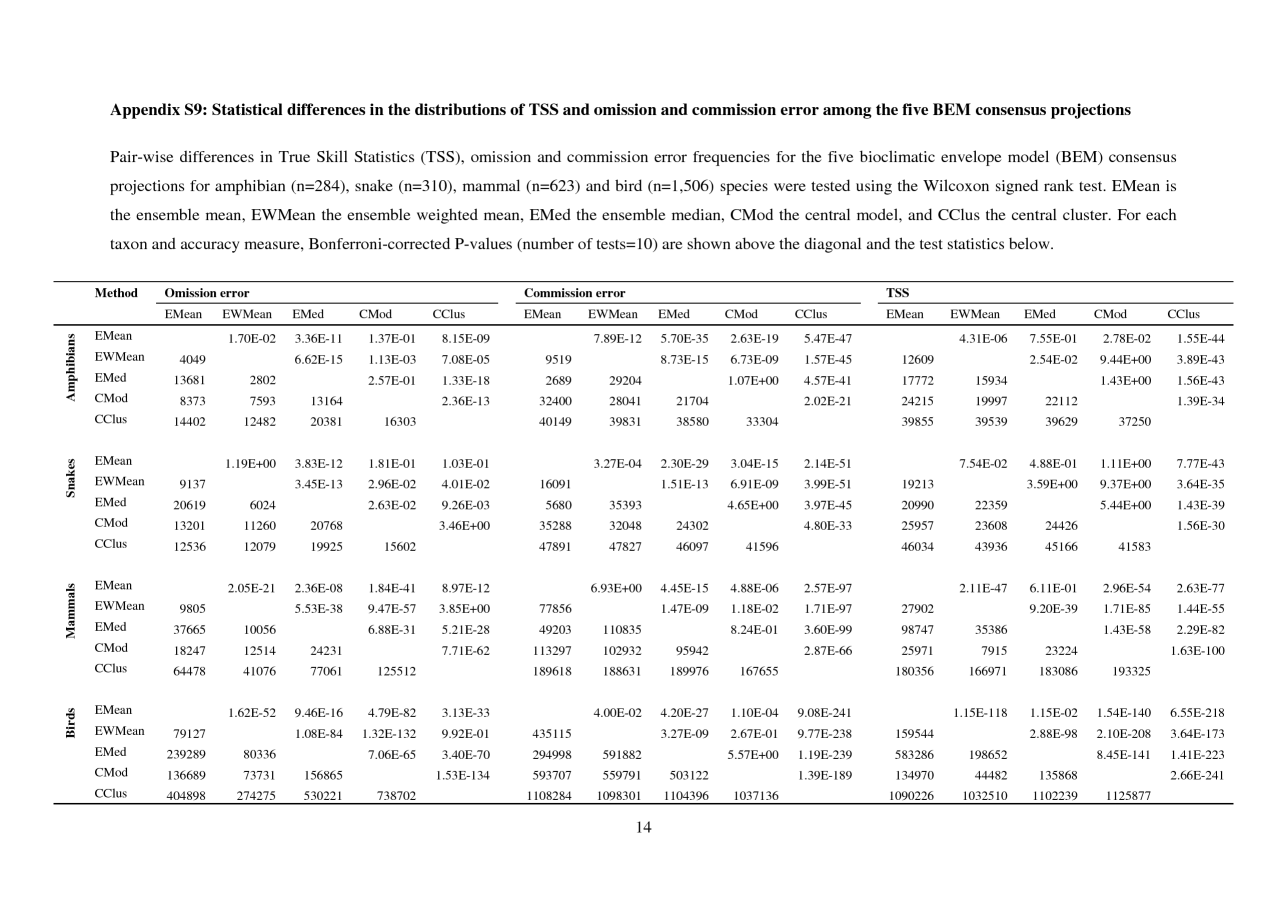

Supplement: Supplementary file 18 [file gcb0018-1253-SD22.png]

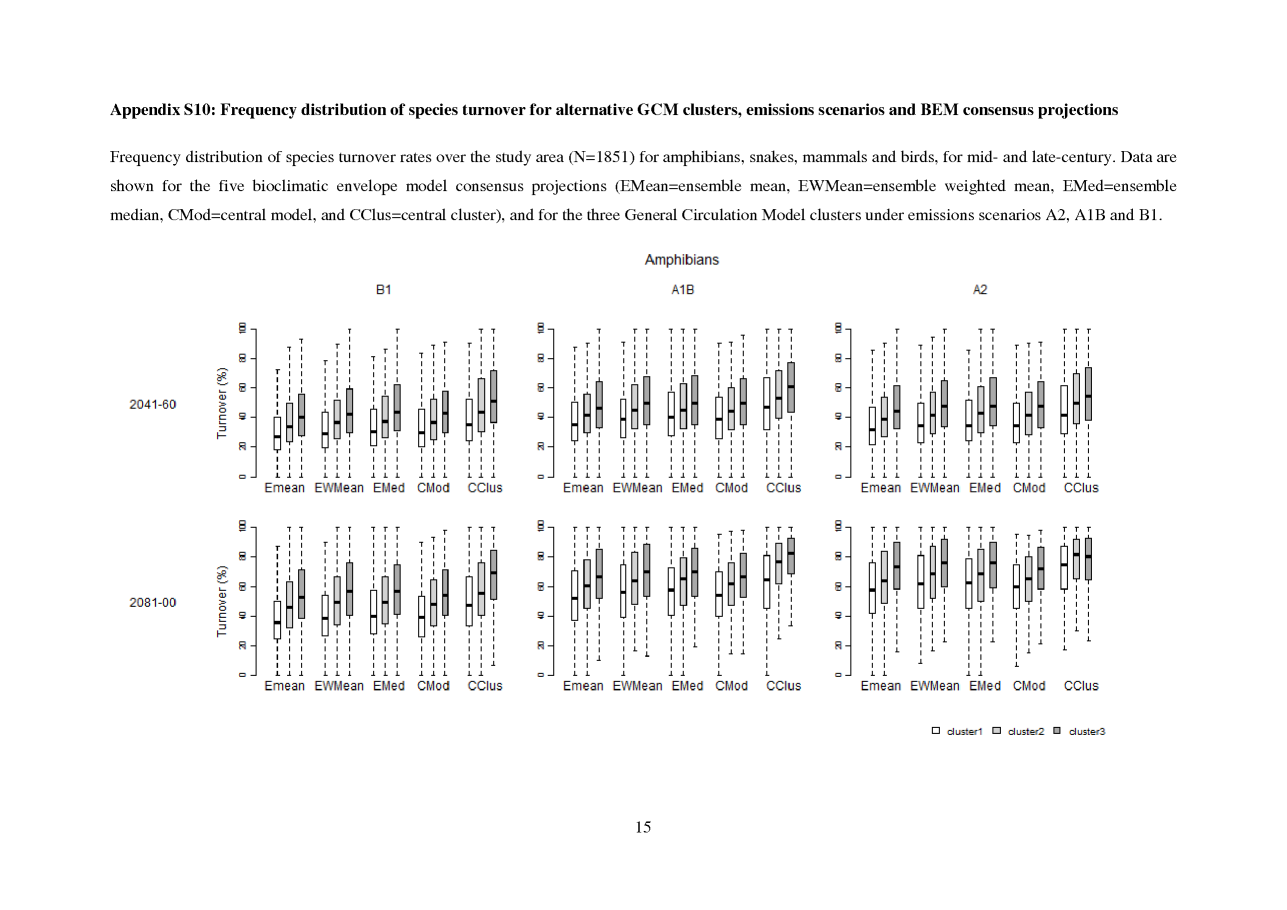

Supplement: Supplementary file 20 [file gcb0018-1253-SD23.png]

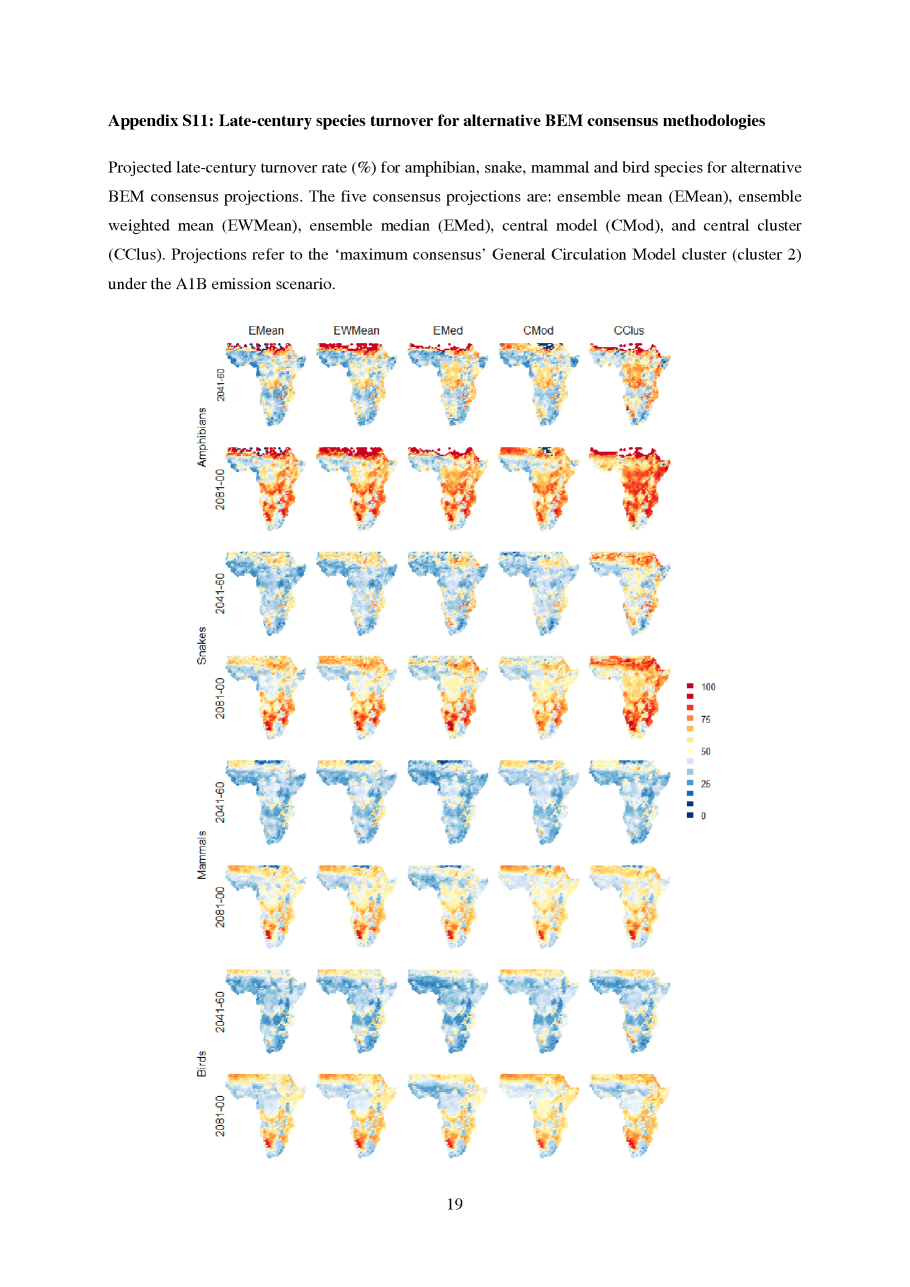

Supplement: Supplementary file 22 [file gcb0018-1253-SD24.png]

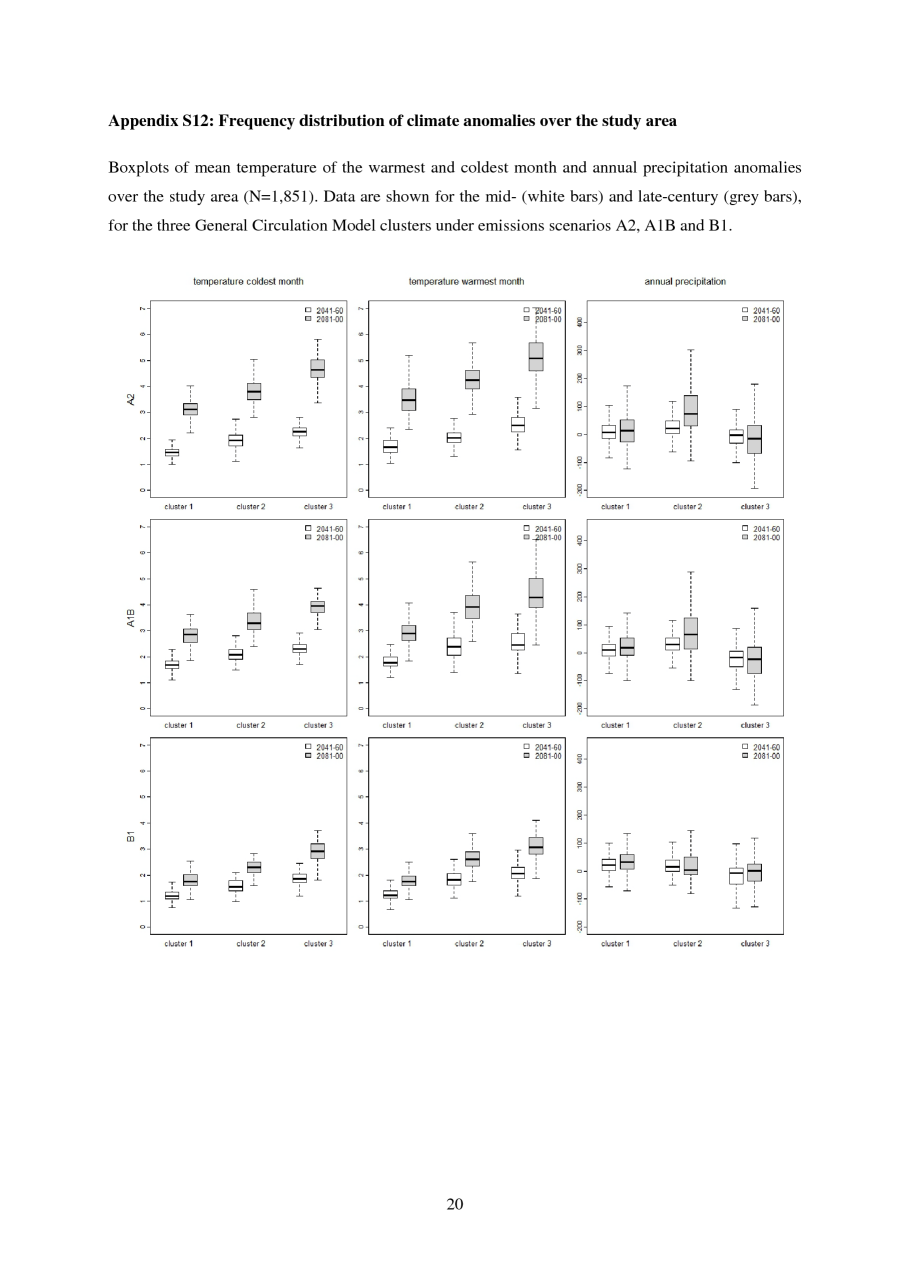

Supplement: Supplementary file 24 [file gcb0018-1253-SD25.png]

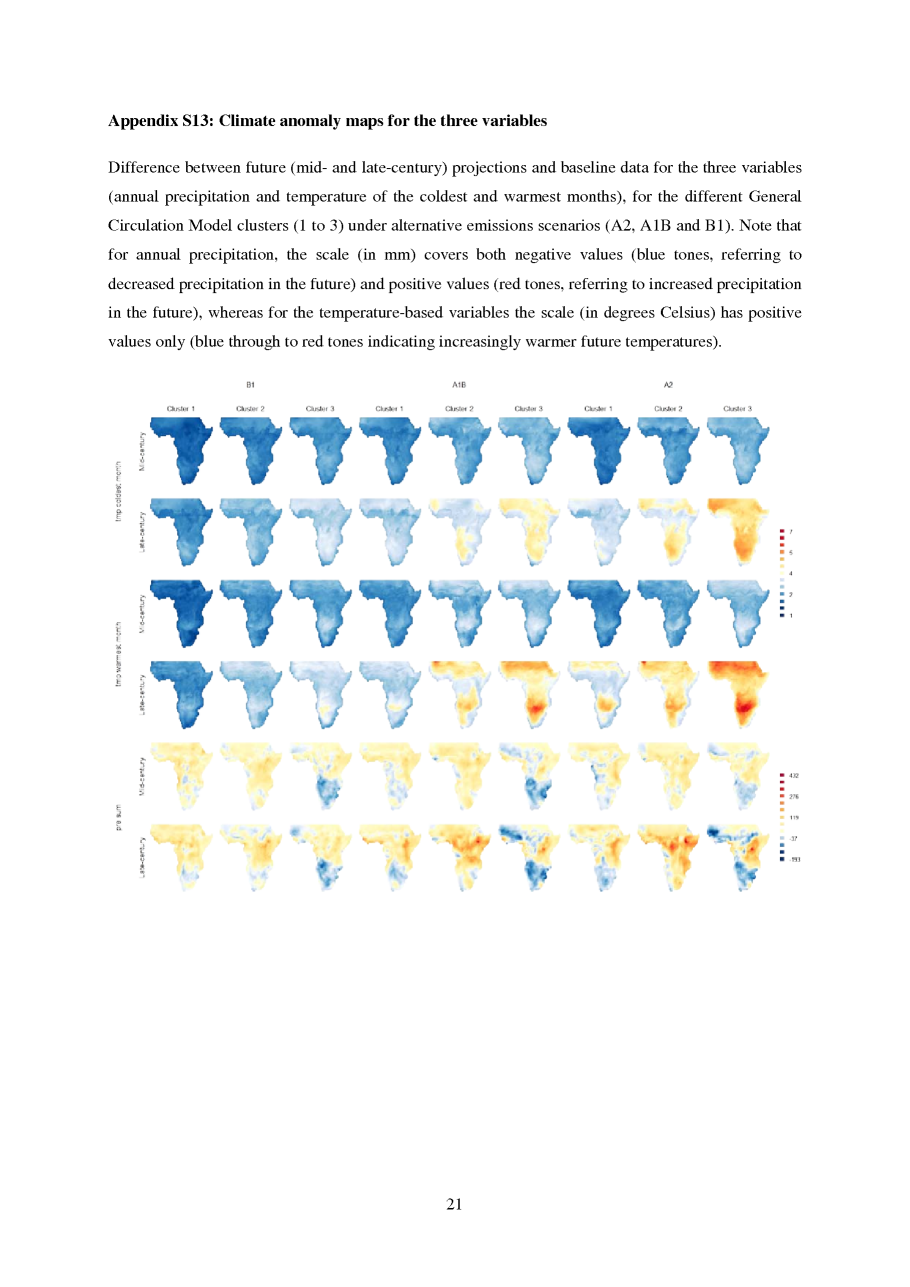

Supplement: Supplementary file 26 [file gcb0018-1253-SD26.png]
