# Supplementary material for: Exploring consensus in 21st century projections of climatically suitable areas for African vertebrates
Source: Glob Chang Biol. 2011 Dec 30;18(4):1253–69. doi: 10.1111/j.1365-2486.2011.02605.x (PMC3597255; doi:10.1111/j.1365-2486.2011.02605.x)
Supplement: Supplementary file 3 [file gcb0018-1253-SD2.pdf]

Appendix S2: General Circulation Models used in the study

17 General Circulation Models (GCM) were used in this study. Their native resolution is shown in the table below, but downscaled projections at 10’ (Tabor & Williams, 2010) were used here. Model name abbreviations used in our study are also shown. The GCM clusters in which each GCM was included are given in the shaded columns for the three emissions scenarios (A2, A1B and B1).

Source	Model	Abbrev.	Resolution		Clusters		
			y	x	A2	A1B	B1
Bjerknes Centre for Climate Research	BCCR-BCM2.0	bc2	2.80	2.80	2	1	3
National Center for Atmospheric Research	CCSM3	cs3	1.41	1.41	2	1	1
Canadian Centre for Climate Modelling and Analysis	CGCM3.1(T47)	t47	3.75	3.75	3	3	3
Centre National de Recherches Météorologiques, Météo-France	CNRM-CM3	cm3	2.80	2.80	3	3	3
Commonwealth Scientific and Industrial Research Organisation (CSIRO) Atmospheric Research	CSIRO-MK3.0	m30	1.90	1.90	1	1	1
Commonwealth Scientific and Industrial Research Organisation (CSIRO) Atmospheric Research	CSIRO-MK3.5	m35	1.90	1.90	3	2	2
Max Planck Institute for Meteorology	ECHAM5/MPI-OM	eh5	1.90	1.90	3	2	2
Meteorological Institute of the University of Bonn, Meteorological Research Institute of the Korea Meteorological Administration (KMA), and Model and Data Group	ECHO-G	ecg	3.90	3.90	2	3	3
Geophysical Fluid Dynamics Laboratory (GFDL), National Oceanic and Atmospheric, Administration (NOAA), U.S. Department of Commerce	GFDL-CM2.0	cm2	2.00	2.50	3	2	3
Geophysical Fluid Dynamics Laboratory (GFDL), National Oceanic and Atmospheric, Administration (NOAA), U.S. Department of Commerce	GFDL-CM2.1	c21	2.00	2.50	3	2	3
Goddard Institute for Space Studies (GISS), National Aeronautics and Space Administration (NASA)	GISS-ER	mer	4.00	5.00	2	3	3
Institute for Numerical Mathematics	INM-CM3.0	im3	4.00	5.00	2	1	3
Institut Pierre Simon Laplace	IPSL-CM4	cm4	2.50	3.75	3	2	2
Center for Climate System Research (University of Tokyo), National Institute for Environmental Studies, and Frontier Research Center for Global Change	MIROC3.2 (medium resolution)	32m	2.80	2.80	1	3	3
Meteorological Research Institute	MRI-CGCM2.3.2	c23	2.80	2.80	1	1	1
National Center for Atmospheric Research	PCM	pc1	2.80	2.80	1	1	1
Hadley Centre for Climate Prediction	UKMO-HadCM3	hd3	2.50	3.75	3	2	2
