# Supplementary material for: Exploring consensus in 21st century projections of climatically suitable areas for African vertebrates
Source: Glob Chang Biol. 2011 Dec 30;18(4):1253–69. doi: 10.1111/j.1365-2486.2011.02605.x (PMC3597255; doi:10.1111/j.1365-2486.2011.02605.x)
Supplement: Supplementary file 5 [file gcb0018-1253-SD3.pdf]

Appendix S3: R scripts used to build the "central cluster" consensus projections

The 'central cluster' consensus methodology investigates patterns of central tendency among groups of co-varying projections. The projections are clustered based on the similarities, for each variable, between each projection and the multi-model median projection. Similarities are defined based on two aggregated point-wise measures of regional deviations among simulations of a given variable: the signed standardised anomaly (*D*) to measure deviation in magnitude and sign, and the spatial pattern Pearson correlation (*R*) to measure agreement in spatial pattern (Duan & Phillips, 2010). If f_x is a given simulation at point *x*, e_x the median of all simulations at point *x*, and *S* the total number of grid points over the study area, then *D* and *R* are given by:

$$D = \frac{\sum \left(\frac{f_x - e_x}{sd(f_x)} \right)}{S} \qquad R = \frac{S\left(\sum f_x - e_x\right) - \sum f_x \times \sum e_x}{\sqrt{S \sum f_x^2 - \sum (f_x)^2 \times \sqrt{S \sum e_x^2 - (\sum e_x)^2}}}$$

This methodology was applied both to the 17 General Circulation Model simulations of the three variables and to the seven bioclimatic envelope model simulations of all species in each taxa. We show below the R scripts used for the General Circulation Models.

Script 1 computes the similarity measures for each variable. **Script 2** performs the clustering on late-century projections. *k-means* was used, with the centres defined by the median points of single linkage hierarchical clusters based on the Euclidean distance matrix (Venables & Ripley, 2003). The significance of the differences between clusters was tested with Anosim, a non-parametric test of analysis of similarity (Clarke & Warwick, 1994). **Script 3** computes the consensus projections for all time periods, i.e. the median of the simulations of a given variable in a cluster.

SCRIPT 1. COMPUTING D AND R

```
sres <- c("A2","A1B","B1")  # emissions scenarios
time <- c("4160","8100")  # time periods
gcm <- c("32m", "bc2", "c21", "c23", "cm2", "cm3", "cm4", "cs3",  "ecg", "eh5", "hd3", "im3", "m30", "m35", "mer", "pc1", "t47")  # general circulation models
variables <- c("tcm","twm","psum")  # variables
# The study area has 1851 points
```

# creating the functions to calculate D and R

```
D <- function(f,e) colSums((f-e)/sd(f))/dim(f)[-2]
R <- function(f,e)  ((dim(f)[-2])*colSums(f*e)-colSums(f)*sum(e))/((sqrt((dim(f)[-2])*colSums(f^2)-colSums(f)^2))*(sqrt((dim(f)[-2])*sum(e^2)-sum(e)^2)))
```

# computing D and R

```
# input data is stored in an array with values for the 3 climatic variables,
# for each emissions scenario and time period
var3gcm <- array(NA, dim = c(1851, length(gcm), length(sres), length(time), length(variables)),
           dimnames = list(NULL, gcm, sres, time, variables))

for (s in 1: length(sres))
  {
  # output data to be saved in arrays
  DR <- array(NA, dim=c(length(gcm), length(variables)*2),
        dimnames = list(gcm, paste(c("D","R"), prefix = rep(variables, each=2))))

  var3 <- var3gcm[, ,s,2,]              # late-century projections are used

  for (v in 1: length(variables))
    {
    med <- apply(var3[, ,v],1,median)
    f <- var3[, ,v]
    e <- med
    Dvalues <- D(f,e)
    Rvalues <- R(f,e)

    DR[ ,grep("D", colnames(DR))] <- Dvalues
    DR[ ,grep("R", colnames(DR))] <- Rvalues
    }
save(DR, file = paste("DR8100", sres[s], sep="_")
  }
```

SCRIPT 2. CLUSTERING

```
library(vegan)
k=3     # number of clusters

for (s in 1: length(sres))
  {
  DR <- get(load(paste("DR8100", sres[s], sep="")))
```

```
  # hierarchical clustering on the D and R distance matrix for all variables
  DR_dist <- get(load(paste("DRdist8100", sres[s], sep="")))
  fith <- hclust(DR_dist, method = "single")

  hcentres <- tapply(DR, list(rep(cutree(fith, k), ncol(DR)), col(DR)), median)
  dimnames(hcentres) <- list(NULL, dimnames(DR)[[2]])

# k-means clustering using the centres from the hierarchical clustering
  set.seed(hcentres)
  fit <- kmeans(DR, k)

  centres <- aggregate(DR, by = list(fit$cluster), FUN=mean)
  save(centres, file = paste("centres_DR_kmeans", k, sres[s], sep=""))
# the centres are used to identify the maximum consensus 'central cluster'

  DR_grouping <- data.frame(DR, fit$cluster)
  rownames(DR_grouping) = gcm
  save(DR_grouping, file = paste("DRgrouping", k, sres[s], sep=""))

# Anosim testing
  DR_ano <- anosim(DR_dist, DR_grouping$fit.cluster, permutations=999)
  DR_anosim[s,1] <- round(DR_ano$statistic, digits=3)
  DR_anosim[s,2] <- round(DR_ano$signif, digits=3)
  }
save(DR_anosim, file= paste("anosim_kmeans_clust",k, sep=""))
```

SCRIPT 3. COMPUTING AVERAGE SIMULATIONS FOR EACH CLUSTER

```
for (s in 1:length(sres))
  {
  DR_grouping <- get(load(paste("DRgrouping", k, sres[s], sep="")))

  for (cl in 1:k)              # k clusters
    {
    clust <- DR_grouping[DR_grouping$fit.cluster == cl,]
    gcmnames <- rownames(clust)
    gcmmednames <- append(gcmnames, "med")
```

```
for (t in 1:2)              # averages computed for the two time periods (baseline and 2081-00)
  {
  clustmed <- array(NA, dim=c(1851, length(variables), length(gcmmednames)),
                    dimnames=list(NULL, variables, gcmmednames))

  for (u in 1:length(gcmnames))   # number of GCMs in the cluster
    {
    for (v in 1:length(variables))
      {
      var3 <- var3gcm[ ,gcmnames[u],s,t,v]
      clustmed[ ,v,u] <- var3

      for (r in 1:1851)
        {
        med <- median(clustmed[r,v,1:length(gcmnames)])
        clustmed[r,v,length(gcmmednames)] <- med
        }
      }
    }
    clustm <- as.matrix(clustmed[, ,length(gcmmednames)])
    save(clustm, file=paste("clust", cl, "_", time[t], sres[s], "_3var", sep=""))
    }
  }
}
```
