# Supplementary material for: Exploring consensus in 21st century projections of climatically suitable areas for African vertebrates
Source: Glob Chang Biol. 2011 Dec 30;18(4):1253–69. doi: 10.1111/j.1365-2486.2011.02605.x (PMC3597255; doi:10.1111/j.1365-2486.2011.02605.x)
Supplement: Supplementary file 7 [file gcb0018-1253-SD4.pdf]

Appendix S4: Sources of uncertainty in the ensemble of forecasts from individual BEMs

A point-wise three-way analysis of variance (ANOVA) without replication was performed for each grid cell over the study area (N=1,851) to evaluate the relative contributions of bioclimatic envelope models (BEM), the General Circulation Model clusters (GCMcons), the emissions scenarios (SRES), and interactions among these factors, to the variability in turnover forecasts for each taxon. The values in the table correspond to the median value and (in brackets) the lower and upper quartiles of the proportion of the total sum of squares attributed to each factor and are shown for both mid- and late-century, for each taxon.

	**Amphibians**		**Snakes**		**Mammals**		**Birds**	
**2041-60**								
BEM	**76.5**	(61.1-87.4)	**81.7**	(70.9-88.9)	**76.2**	(61.8-86.4)	**79.8**	(61.7-87.9)
GCMcons	**7.3**	(2.7-16.2)	**6.8**	(3.0-13.4)	**7.6**	(3.7-16.5)	**8.2**	(4.2-19.1)
SRES	**3.3**	(1.3-6.7)	**3.1**	(1.6-5.6)	**4.0**	(2.1-7.6)	**4.0**	(2.2-7.7)
BEM:GCMcons	**3.2**	(1.6-5.8)	**2.2**	(1.3-4.1)	**2.8**	(1.5-5.4)	**2.2**	(1.2-4.3)
BEM:SRES	**1.4**	(0.8-2.6)	**1.1**	(0.6-1.8)	**1.2**	(0.7-2.2)	**1.0**	(0.5-1.7)
GCMcons:SRES	**1.1**	(0.4-2.2)	**0.9**	(0.4-1.7)	**1.0**	(0.5-2.2)	**0.9**	(0.4-1.9)
BEM:GCMcons:SRES	**2.2**	(1.2-4.0)	**1.6**	(0.9-2.8)	**1.8**	(1-3.4.0)	**1.2**	(0.6-2.1)
**2081-00**								
BEM	**63.4**	(38.7-83.1)	**69.0**	(48.2-82.5)	**60.6**	(37.3-76.6)	**62.6**	(34.1-76.7)
GCMcons	**7.4**	(2.6-16.2)	**7.5**	(4.0-14.1)	**9.6**	(5.1-16.4)	**10.2**	(6.2-18.2)
SRES	**12.0**	(3.8-30.9)	**13.5**	(6.1-27.6)	**18.2**	(9.5-33.7)	**17.0**	(9.1-35.2)
BEM:GCMcons	**2.4**	(1.4-3.9)	**1.7**	(1.1-2.6)	**1.9**	(1.2-3.2)	**1.8**	(1.1-2.7)
BEM:SRES	**2.5**	(1.5-4.3)	**2.0**	(1.2-3.1)	**2.3**	(1.4-3.8)	**2.2**	(1.4-3.6)
GCMcons:SRES	**1.4**	(0.5-2.8)	**0.9**	(0.4-2.0)	**1.1**	(0.5-2.3)	**1.0**	(0.5-2.4)
BEM:GCMcons:SRES	**2.4**	(1.4-4.2)	**1.7**	(1.1-2.8)	**1.8**	(1.1-3.0)	**1.4**	(0.9-2.3)
