# Supplementary material for: Exploring consensus in 21st century projections of climatically suitable areas for African vertebrates
Source: Glob Chang Biol. 2011 Dec 30;18(4):1253–69. doi: 10.1111/j.1365-2486.2011.02605.x (PMC3597255; doi:10.1111/j.1365-2486.2011.02605.x)

Appendix S5: Non-analogue climate maps

For both mid- and late-century, non-analogue climates correspond to the sum of the areas where mean temperature of the warmest month, mean temperature of the coldest month and annual precipitation values beyond the observed ranges in the baseline period are projected. Areas in red were projected to experience future climate conditions lacking baseline analogues. The percentage of the total area projected to experience non-analogue climates is indicated in red and the same percentage for each variable (tcm=temperature of the coldest month, tw=temperature of the warmest month, ps=annual precipitation) in black underneath each map. Data are shown for the three General Circulation Model clusters under each emissions scenario (A2, A1B and B1).

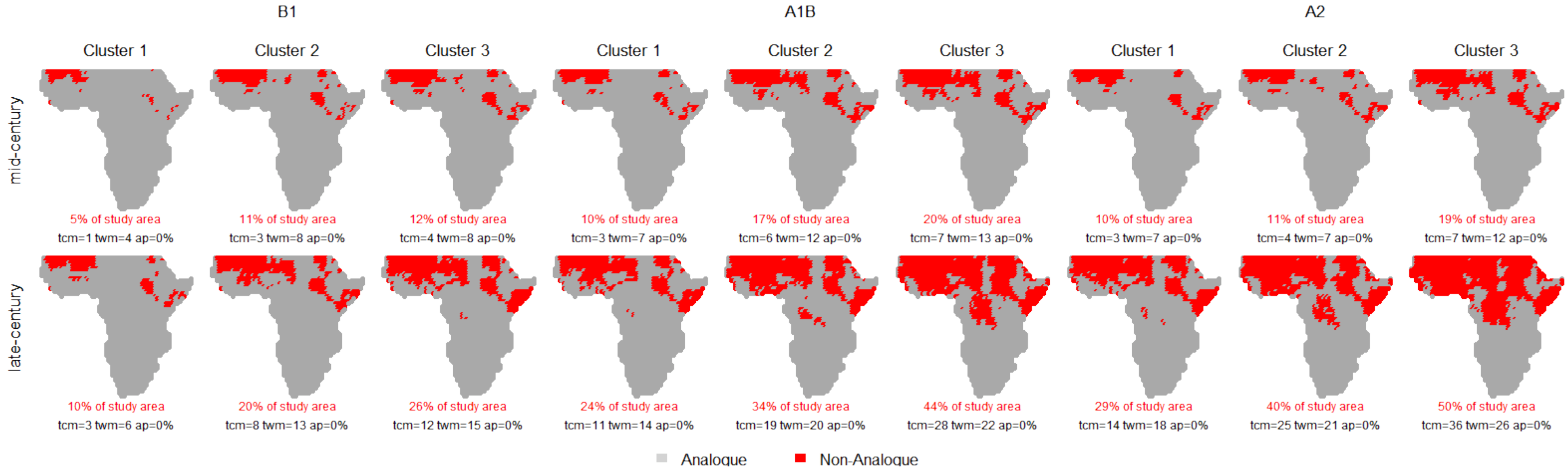

Supplement: Supplementary file 9 [file gcb0018-1253-SD5.pdf]
