# Supplementary material for: Exploring consensus in 21st century projections of climatically suitable areas for African vertebrates
Source: Glob Chang Biol. 2011 Dec 30;18(4):1253–69. doi: 10.1111/j.1365-2486.2011.02605.x (PMC3597255; doi:10.1111/j.1365-2486.2011.02605.x)
Supplement: Supplementary file 11 [file gcb0018-1253-SD6.pdf]

Appendix S6: Single-BEMs selected for the 'central clusters' and Anosim results

For each taxon and climate projection, the seven bioclimatic envelope models were clustered based on similarities in magnitude and spatial pattern between single-model probabilistic projections and the multi-model median probabilistic projection for late-century. The *k-means* clustering resulted in four clusters, shown below for the three General Circulation Model clusters under each emission scenario (A2, A1B and B1). Cluster 1 is the maximum consensus 'central cluster'. The analysis of similarity (Anosim) test was used to test the statistical significance of the clustering.

			BEM clusters							Anosim	
	**SRES**	**GCM**	**ANN**	**GAM**	**GBM**	**GLM**	**MARS**	**FDA**	**RF**	**Statistics**	**P-value**
**Amphibians**	**A2**	Cluster 1	4	2	1	1	3	3	3	0.912	0.004
		Cluster 2	4	2	1	1	3	3	3	0.765	0.013
		Cluster 3	2	4	1	1	3	3	1	0.971	0.004
	**A1B**	Cluster 1	4	2	1	2	1	3	1	0.853	0.004
		Cluster 2	4	2	1	1	3	3	3	0.853	0.004
		Cluster 3	2	4	1	1	3	3	3	0.735	0.027
	**B1**	Cluster 1	4	2	1	1	3	3	3	0.824	0.008
		Cluster 2	4	2	1	2	1	3	1	0.853	0.004
		Cluster 3	4	2	1	1	3	3	3	0.912	0.004
**Snakes**	**A2**	Cluster 1	3	2	1	1	4	4	4	0.853	0.004
		Cluster 2	2	4	1	1	3	3	3	0.882	0.004
		Cluster 3	2	4	1	1	3	3	1	0.941	0.004
	**A1B**	Cluster 1	3	2	1	1	4	4	4	0.853	0.004
		Cluster 2	2	4	1	1	3	3	3	0.853	0.004
		Cluster 3	2	4	1	1	3	3	3	0.882	0.004
	**B1**	Cluster 1	3	2	1	1	4	4	4	0.824	0.002
		Cluster 2	3	2	1	1	4	4	4	0.853	0.004
		Cluster 3	3	2	1	1	4	4	4	0.882	0.004
**Mammals**	**A2**	Cluster 1	4	1	2	1	2	3	2	0.853	0.003
		Cluster 2	4	1	2	1	3	2	2	0.941	0.003
		Cluster 3	4	2	2	1	3	2	2	0.912	0.003
	**A1B**	Cluster 1	4	1	2	1	2	3	2	0.794	0.006
		Cluster 2	4	1	2	1	2	3	2	0.618	0.045
		Cluster 3	4	1	1	2	3	3	1	1.000	0.003
	**B1**	Cluster 1	4	1	2	1	2	3	2	0.933	0.034
		Cluster 2	4	1	2	1	2	3	2	0.765	0.016

	SRES	GCM	BEM clusters							Anosim	
			ANN	GAM	GBM	GLM	MARS	FDA	RF	Statistics	P-value
		Cluster 3	4	1	2	1	2	3	2	1.000	0.003
**Birds**	**A2**	Cluster 1	2	1	1	1	4	3	1	0.933	0.032
		Cluster 2	2	1	1	1	4	3	1	0.911	0.032
		Cluster 3	2	1	1	1	4	3	1	1.000	0.003
	**A1B**	Cluster 1	2	1	1	1	4	3	1	0.978	0.032
		Cluster 2	2	1	1	1	4	3	1	0.933	0.032
		Cluster 3	2	1	1	1	4	3	1	0.882	0.011
	**B1**	Cluster 1	4	1	2	1	2	3	2	0.911	0.032
		Cluster 2	2	1	4	1	4	3	4	0.889	0.032
		Cluster 3	2	1	1	1	4	3	1	0.933	0.032
