# Supplementary material for: Exploring consensus in 21st century projections of climatically suitable areas for African vertebrates
Source: Glob Chang Biol. 2011 Dec 30;18(4):1253–69. doi: 10.1111/j.1365-2486.2011.02605.x (PMC3597255; doi:10.1111/j.1365-2486.2011.02605.x)

Appendix S7: Level of consensus among all BEMs and 'central cluster' BEMs

To evaluate the degree of consensus achieved in bioclimatic envelope model (BEM) consensus projections, Principal Components Analysis (PCA) was performed for each species on the probabilistic projections. The proportion of variance explained by the first principal component axis reflects the degree of consensus among projections. PCAs were performed on baseline and late-century projections, both on the seven single-BEMs and on the 'central cluster' BEMs. The graphs show the frequency distributions across all amphibian (n=284), snake (n=310), mammal (n=623) and bird (n=1,506) species, with the degree of consensus increasing on a scale from 0 to 1. For future projections calculations, the 'maximum consensus' General Circulation Model cluster (cluster 2, the 'central cluster') under the emissions scenario A1B was used.

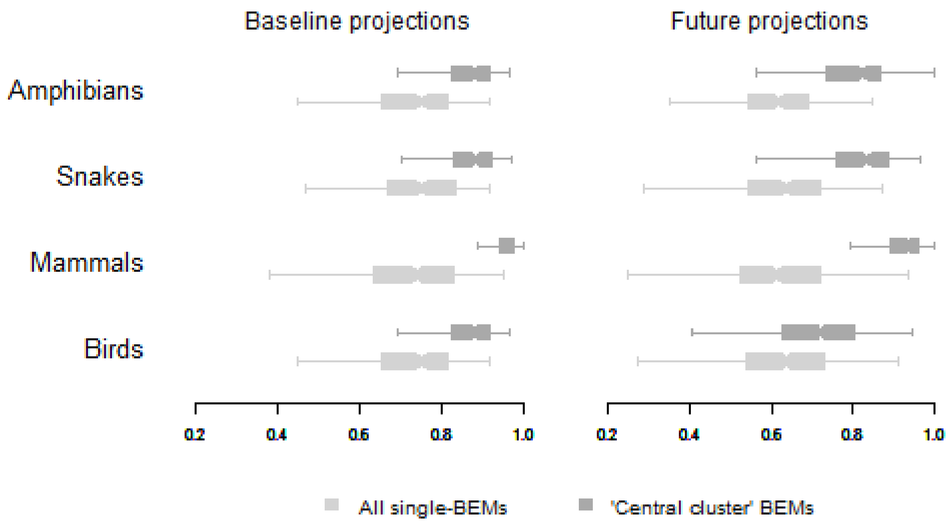

Supplement: Supplementary file 13 [file gcb0018-1253-SD7.pdf]
