# Supplementary material for: Exploring consensus in 21st century projections of climatically suitable areas for African vertebrates
Source: Glob Chang Biol. 2011 Dec 30;18(4):1253–69. doi: 10.1111/j.1365-2486.2011.02605.x (PMC3597255; doi:10.1111/j.1365-2486.2011.02605.x)

Appendix S8: Single-BEMs selected as 'central model' across species

With the 'central model' methodology to combine the ensembles of bioclimatic envelope models (BEM), we selected the model summarising the highest amount of variation among projections for each species. A Principal Components Analysis (PCA) was performed for each species on the late-century projected probabilities and the 'central model' corresponded to the one with the highest PCA loading in the first (consensus) axis. The graphs show the percentage of species for which each of the seven single BEMs was selected as the 'central model'. Results are shown for the projections built with 100% of the data.

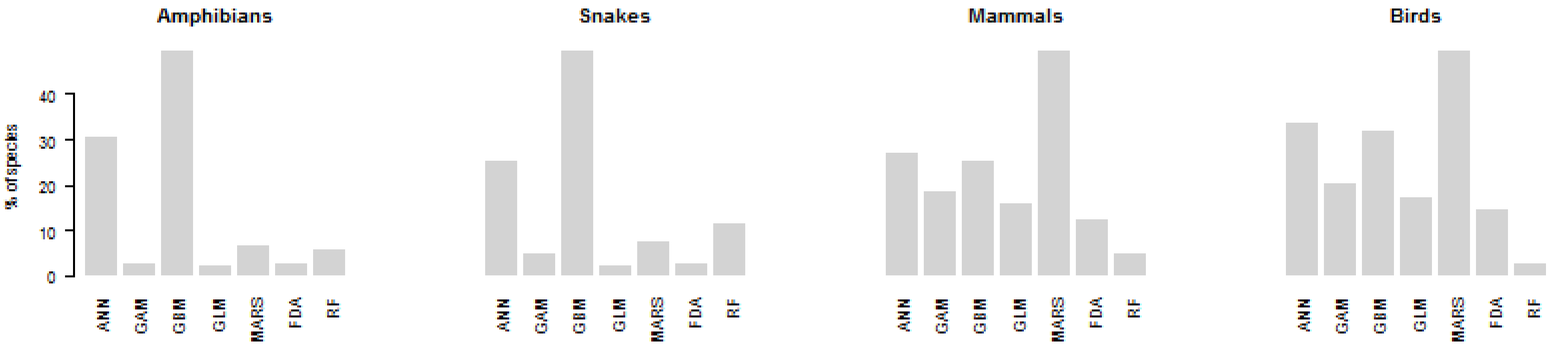

Supplement: Supplementary file 15 [file gcb0018-1253-SD8.pdf]
