# Supplementary material for: Exploring consensus in 21st century projections of climatically suitable areas for African vertebrates
Source: Glob Chang Biol. 2011 Dec 30;18(4):1253–69. doi: 10.1111/j.1365-2486.2011.02605.x (PMC3597255; doi:10.1111/j.1365-2486.2011.02605.x)
Supplement: Supplementary file 17 [file gcb0018-1253-SD9.pdf]

Appendix S9: Statistical differences in the distributions of TSS and omission and commission error among the five BEM consensus projections

Pair-wise differences in True Skill Statistics (TSS), omission and commission error frequencies for the five bioclimatic envelope model (BEM) consensus projections for amphibian (n=284), snake (n=310), mammal (n=623) and bird (n=1,506) species were tested using the Wilcoxon signed rank test. EMean is the ensemble mean, EWMean the ensemble weighted mean, EMed the ensemble median, CMod the central model, and CClus the central cluster. For each taxon and accuracy measure, Bonferroni-corrected P-values (number of tests=10) are shown above the diagonal and the test statistics below.

	**Method**	**Omission error**					**Commission error**					**TSS**				
		EMean	EWMean	EMed	CMod	CClus	EMean	EWMean	EMed	CMod	CClus	EMean	EWMean	EMed	CMod	CClus
**Amphibians**	EMean		1.70E-02	3.36E-11	1.37E-01	8.15E-09		7.89E-12	5.70E-35	2.63E-19	5.47E-47		4.31E-06	7.55E-01	2.78E-02	1.55E-44
	EWMean	4049		6.62E-15	1.13E-03	7.08E-05	9519		8.73E-15	6.73E-09	1.57E-45	12609		2.54E-02	9.44E+00	3.89E-43
	EMed	13681	2802		2.57E-01	1.33E-18	2689	29204		1.07E+00	4.57E-41	17772	15934		1.43E+00	1.56E-43
	CMod	8373	7593	13164		2.36E-13	32400	28041	21704		2.02E-21	24215	19997	22112		1.39E-34
	CClus	14402	12482	20381	16303		40149	39831	38580	33304		39855	39539	39629	37250	
**Snakes**	EMean		1.19E+00	3.83E-12	1.81E-01	1.03E-01		3.27E-04	2.30E-29	3.04E-15	2.14E-51		7.54E-02	4.88E-01	1.11E+00	7.77E-43
	EWMean	9137		3.45E-13	2.96E-02	4.01E-02	16091		1.51E-13	6.91E-09	3.99E-51	19213		3.59E+00	9.37E+00	3.64E-35
	EMed	20619	6024		2.63E-02	9.26E-03	5680	35393		4.65E+00	3.97E-45	20990	22359		5.44E+00	1.43E-39
	CMod	13201	11260	20768		3.46E+00	35288	32048	24302		4.80E-33	25957	23608	24426		1.56E-30
	CClus	12536	12079	19925	15602		47891	47827	46097	41596		46034	43936	45166	41583	
**Mammals**	EMean		2.05E-21	2.36E-08	1.84E-41	8.97E-12		6.93E+00	4.45E-15	4.88E-06	2.57E-97		2.11E-47	6.11E-01	2.96E-54	2.63E-77
	EWMean	9805		5.53E-38	9.47E-57	3.85E+00	77856		1.47E-09	1.18E-02	1.71E-97	27902		9.20E-39	1.71E-85	1.44E-55
	EMed	37665	10056		6.88E-31	5.21E-28	49203	110835		8.24E-01	3.60E-99	98747	35386		1.43E-58	2.29E-82
	CMod	18247	12514	24231		7.71E-62	113297	102932	95942		2.87E-66	25971	7915	23224		1.63E-100
	CClus	64478	41076	77061	125512		189618	188631	189976	167655		180356	166971	183086	193325	
**Birds**	EMean		1.62E-52	9.46E-16	4.79E-82	3.13E-33		4.00E-02	4.20E-27	1.10E-04	9.08E-241		1.15E-118	1.15E-02	1.54E-140	6.55E-218
	EWMean	79127		1.08E-84	1.32E-132	9.92E-01	435115		3.27E-09	2.67E-01	9.77E-238	159544		2.88E-98	2.10E-208	3.64E-173
	EMed	239289	80336		7.06E-65	3.40E-70	294998	591882		5.57E+00	1.19E-239	583286	198652		8.45E-141	1.41E-223
	CMod	136689	73731	156865		1.53E-134	593707	559791	503122		1.39E-189	134970	44482	135868		2.66E-241
	CClus	404898	274275	530221	738702		1108284	1098301	1104396	1037136		1090226	1032510	1102239	1125877	
