# Supplementary material for: Exploring consensus in 21st century projections of climatically suitable areas for African vertebrates
Source: Glob Chang Biol. 2011 Dec 30;18(4):1253–69. doi: 10.1111/j.1365-2486.2011.02605.x (PMC3597255; doi:10.1111/j.1365-2486.2011.02605.x)
Supplement: Supplementary file 19 [file gcb0018-1253-SD10.pdf]

Appendix S10: Frequency distribution of species turnover for alternative GCM clusters, emissions scenarios and BEM consensus projections

Frequency distribution of species turnover rates over the study area (N=1851) for amphibians, snakes, mammals and birds, for mid- and late-century. Data are shown for the five bioclimatic envelope model consensus projections (EMean=ensemble mean, EWMean=ensemble weighted mean, EMed=ensemble median, CMod=central model, and CClus=central cluster), and for the three General Circulation Model clusters under emissions scenarios A2, A1B and B1.

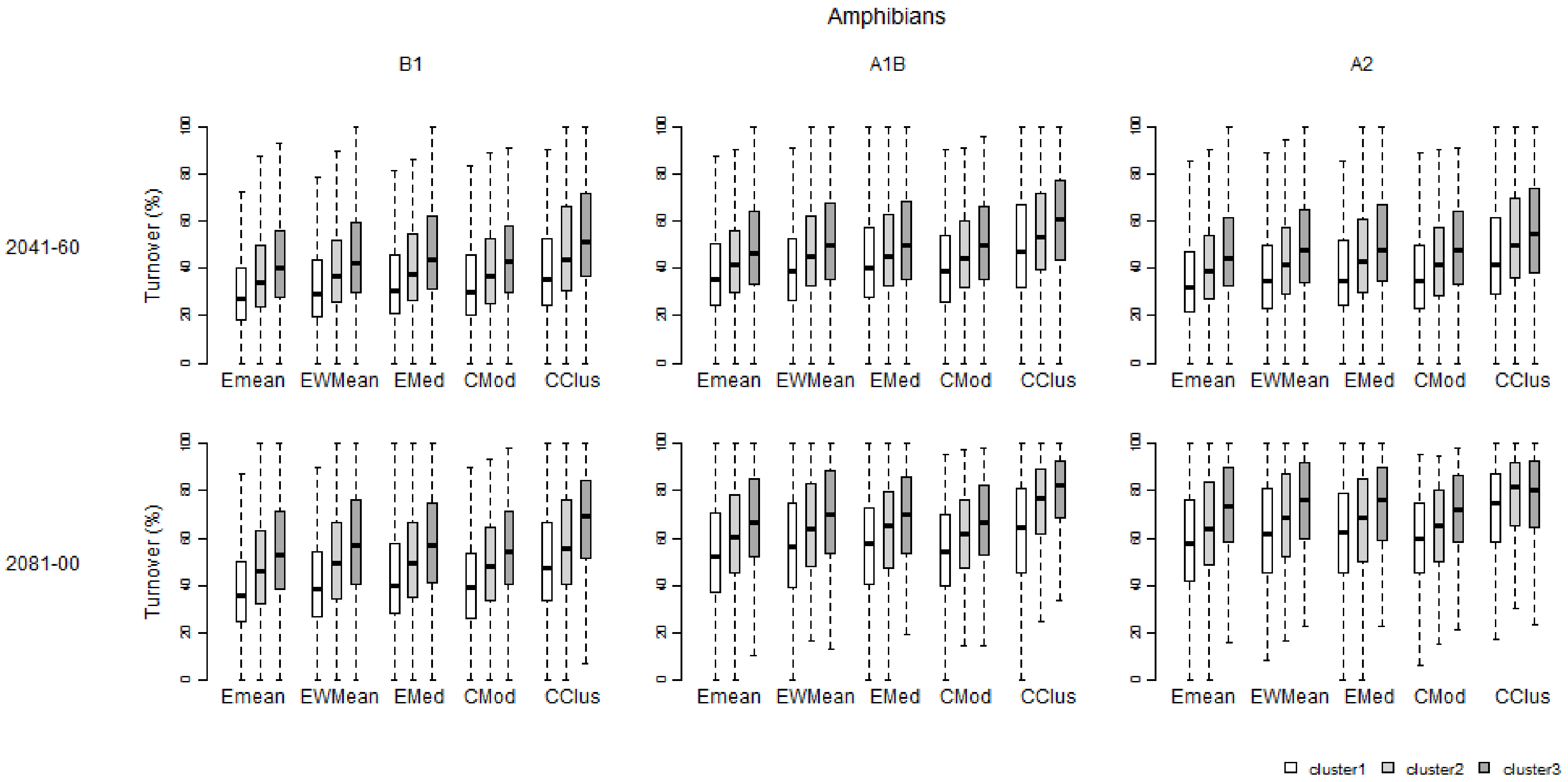

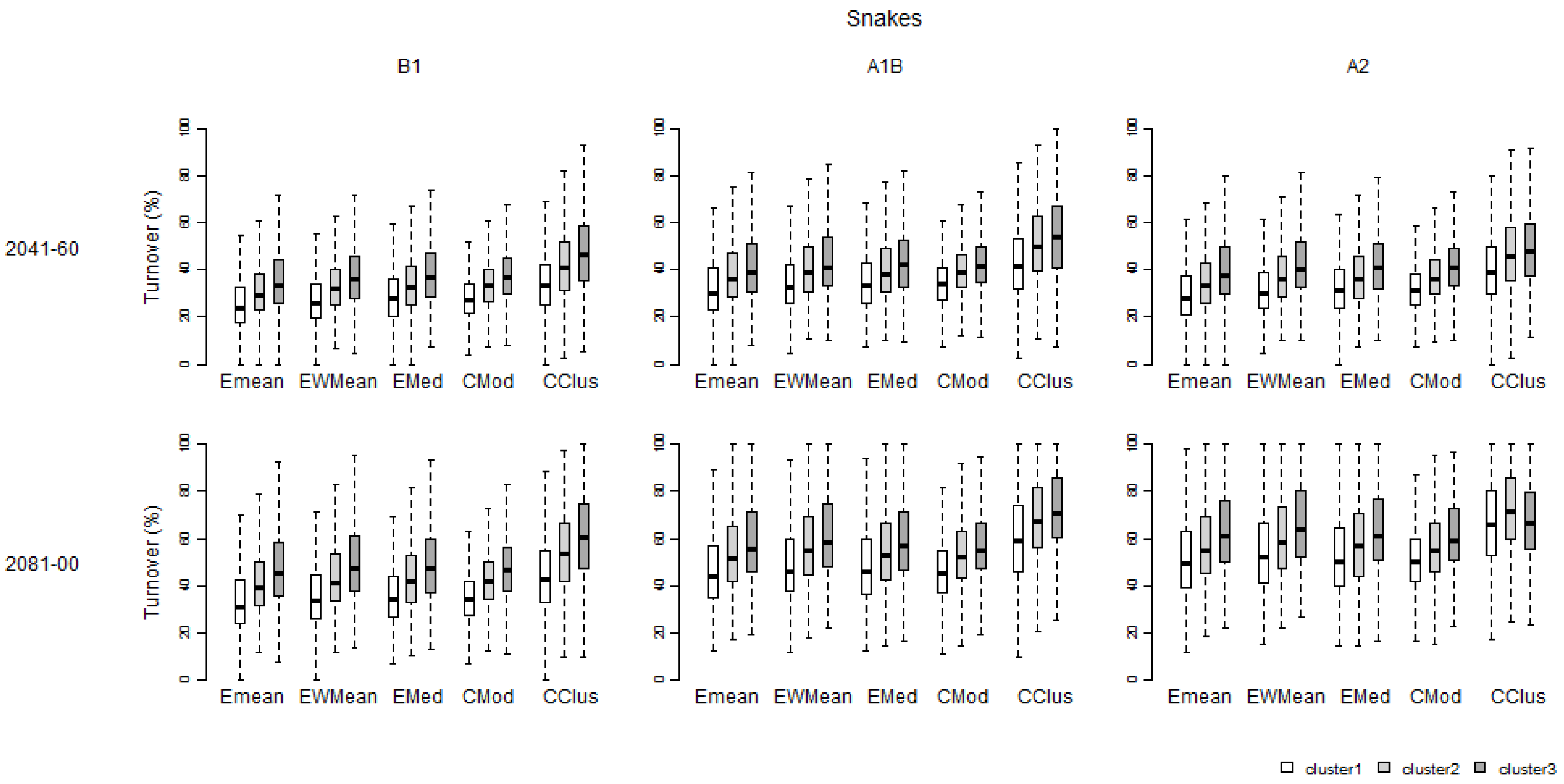
Snakes
B1
A1B
A2
2041-60
2081-00
Turnover (%)
Emean
EWMean
EMed
CMod
CClus
cluster1
cluster2
cluster3

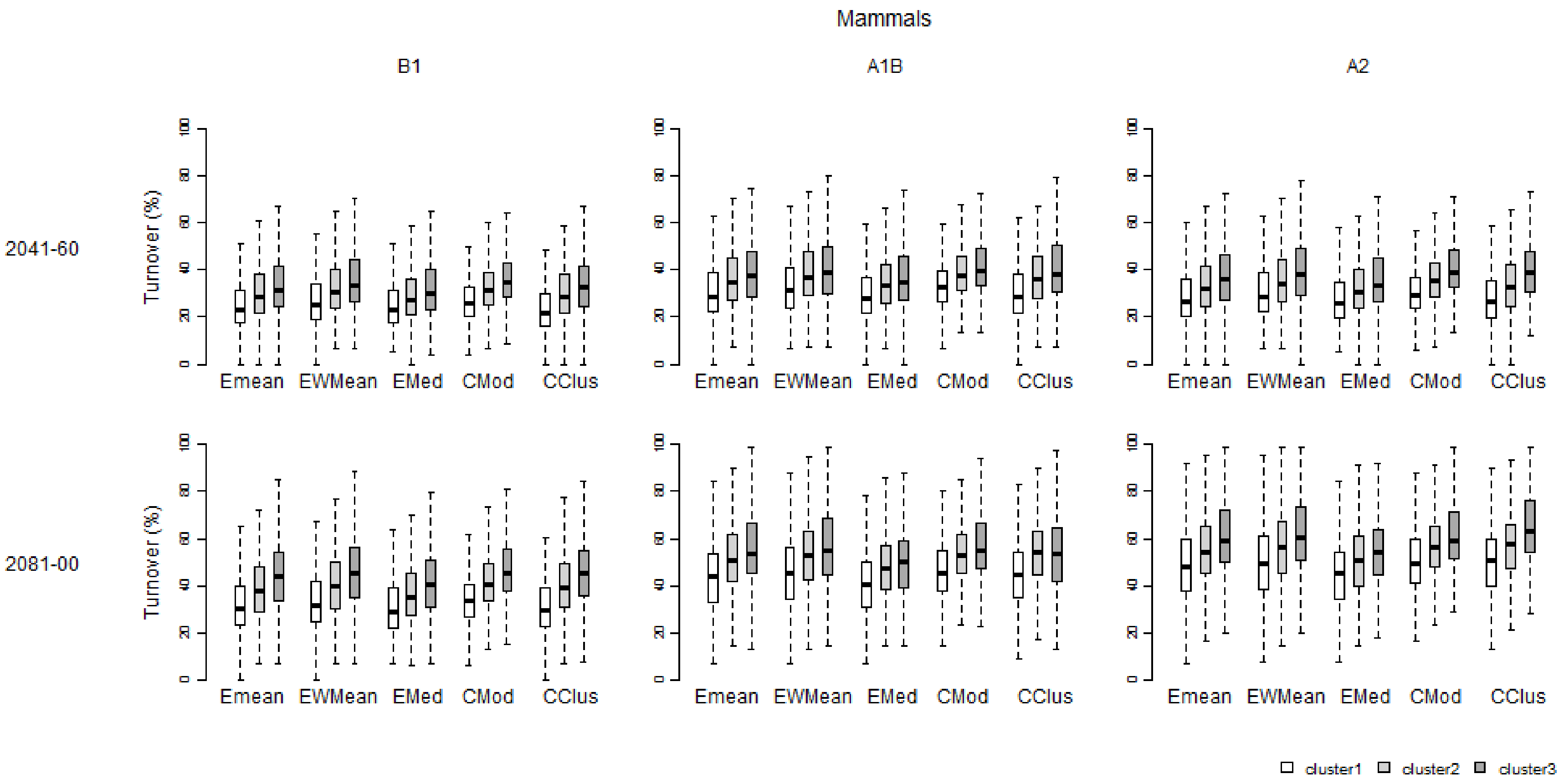
Mammals
B1
A1B
A2
2041-60
2081-00
Turnover (%)
Emean
EWMean
EMed
CMod
CClus
cluster1
cluster2
cluster3

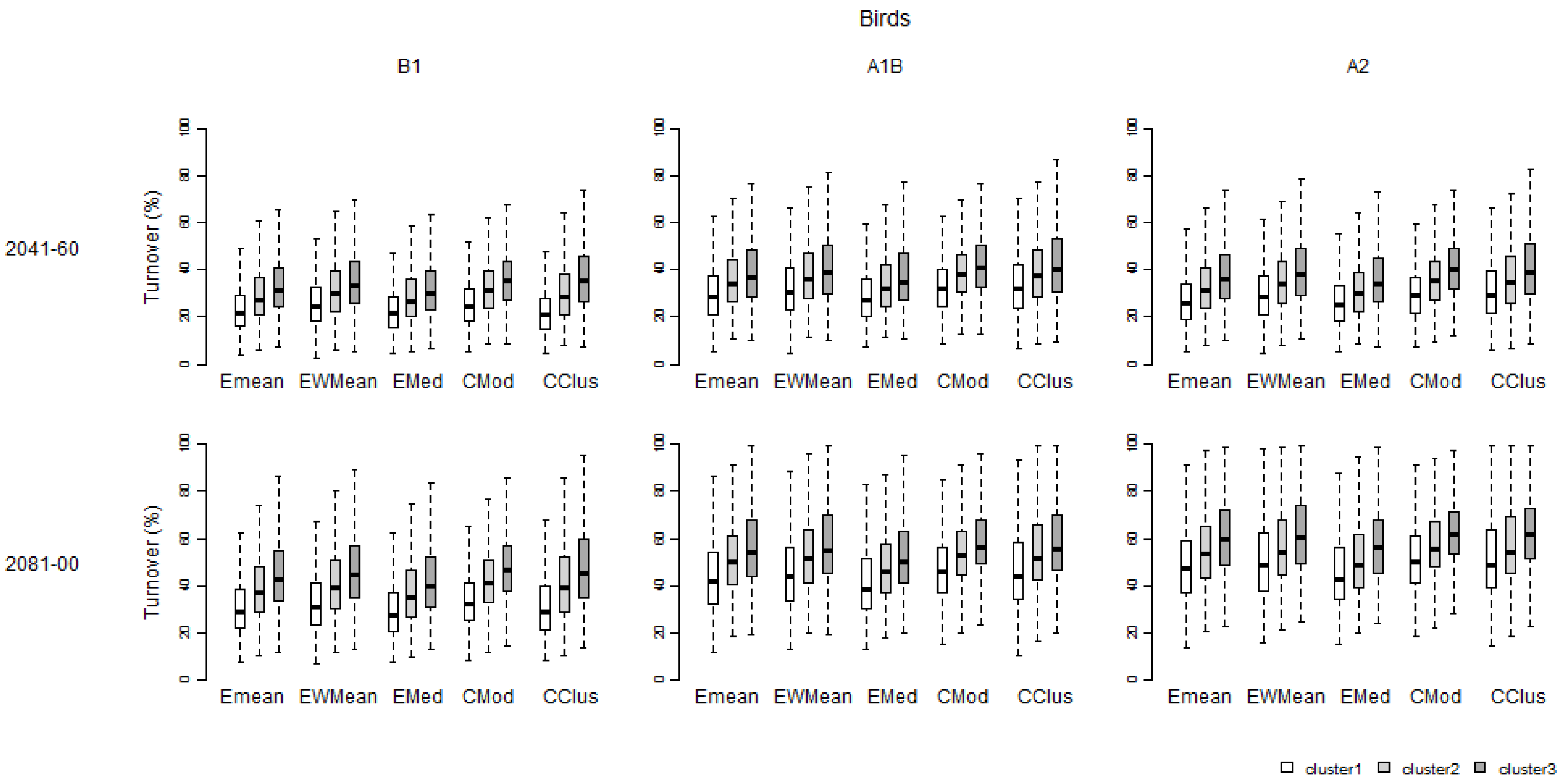
Birds
B1
A1B
A2
2041-60
2081-00
Turnover (%)
Emean
EWMean
EMed
CMod
CClus
cluster1
cluster2
cluster3
