# Supplementary material for: Exploring consensus in 21st century projections of climatically suitable areas for African vertebrates
Source: Glob Chang Biol. 2011 Dec 30;18(4):1253–69. doi: 10.1111/j.1365-2486.2011.02605.x (PMC3597255; doi:10.1111/j.1365-2486.2011.02605.x)

Appendix S11: Late-century species turnover for alternative BEM consensus methodologies

Projected late-century turnover rate (%) for amphibian, snake, mammal and bird species for alternative BEM consensus projections. The five consensus projections are: ensemble mean (EMean), ensemble weighted mean (EWMean), ensemble median (EMed), central model (CMod), and central cluster (CClus). Projections refer to the ‘maximum consensus’ General Circulation Model cluster (cluster 2) under the A1B emission scenario.

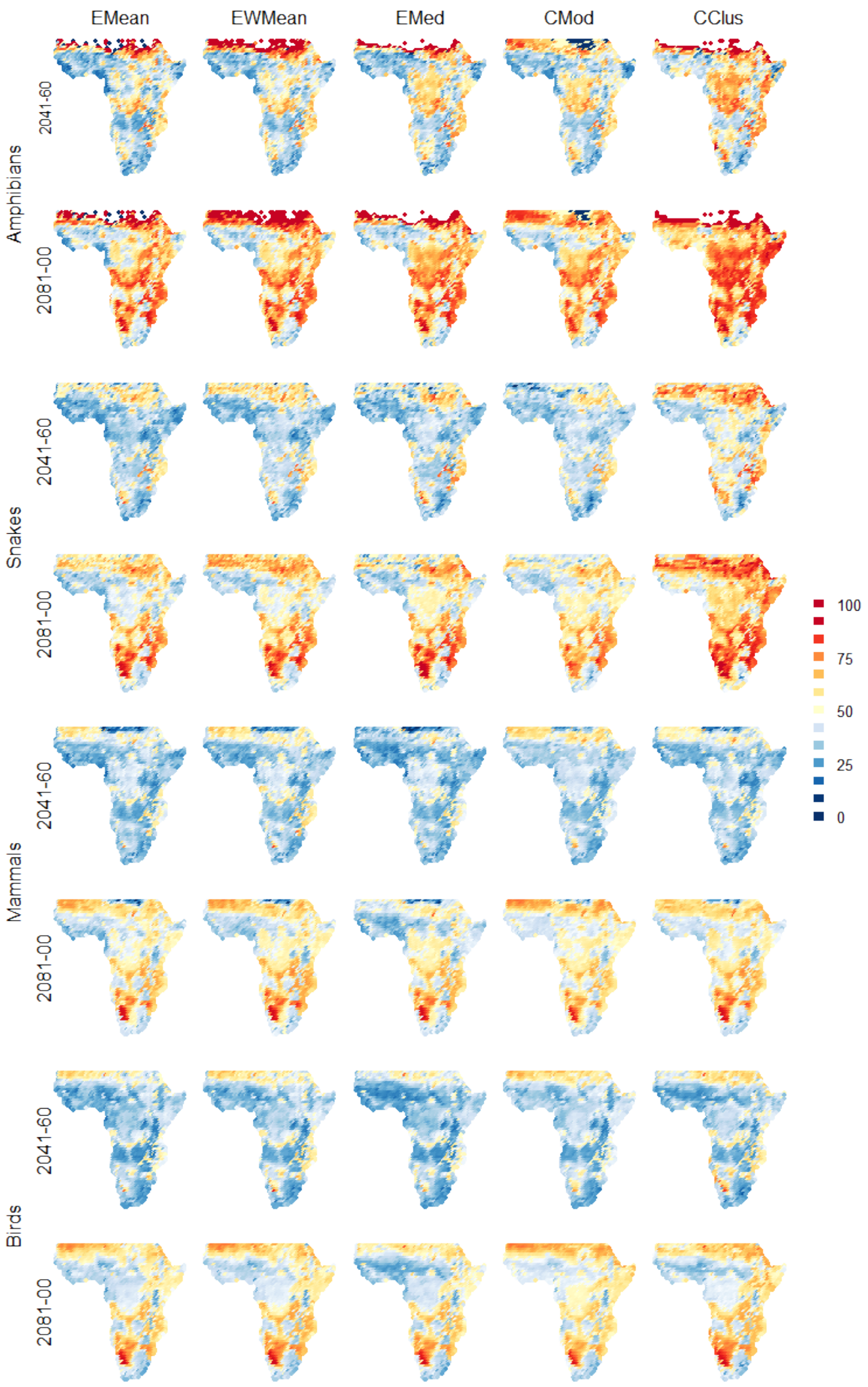

Supplement: Supplementary file 21 [file gcb0018-1253-SD11.pdf]
