# Supplementary material for: Exploring consensus in 21st century projections of climatically suitable areas for African vertebrates
Source: Glob Chang Biol. 2011 Dec 30;18(4):1253–69. doi: 10.1111/j.1365-2486.2011.02605.x (PMC3597255; doi:10.1111/j.1365-2486.2011.02605.x)

Appendix S12: Frequency distribution of climate anomalies over the study area

Boxplots of mean temperature of the warmest and coldest month and annual precipitation anomalies over the study area (N=1,851). Data are shown for the mid- (white bars) and late-century (grey bars), for the three General Circulation Model clusters under emissions scenarios A2, A1B and B1.

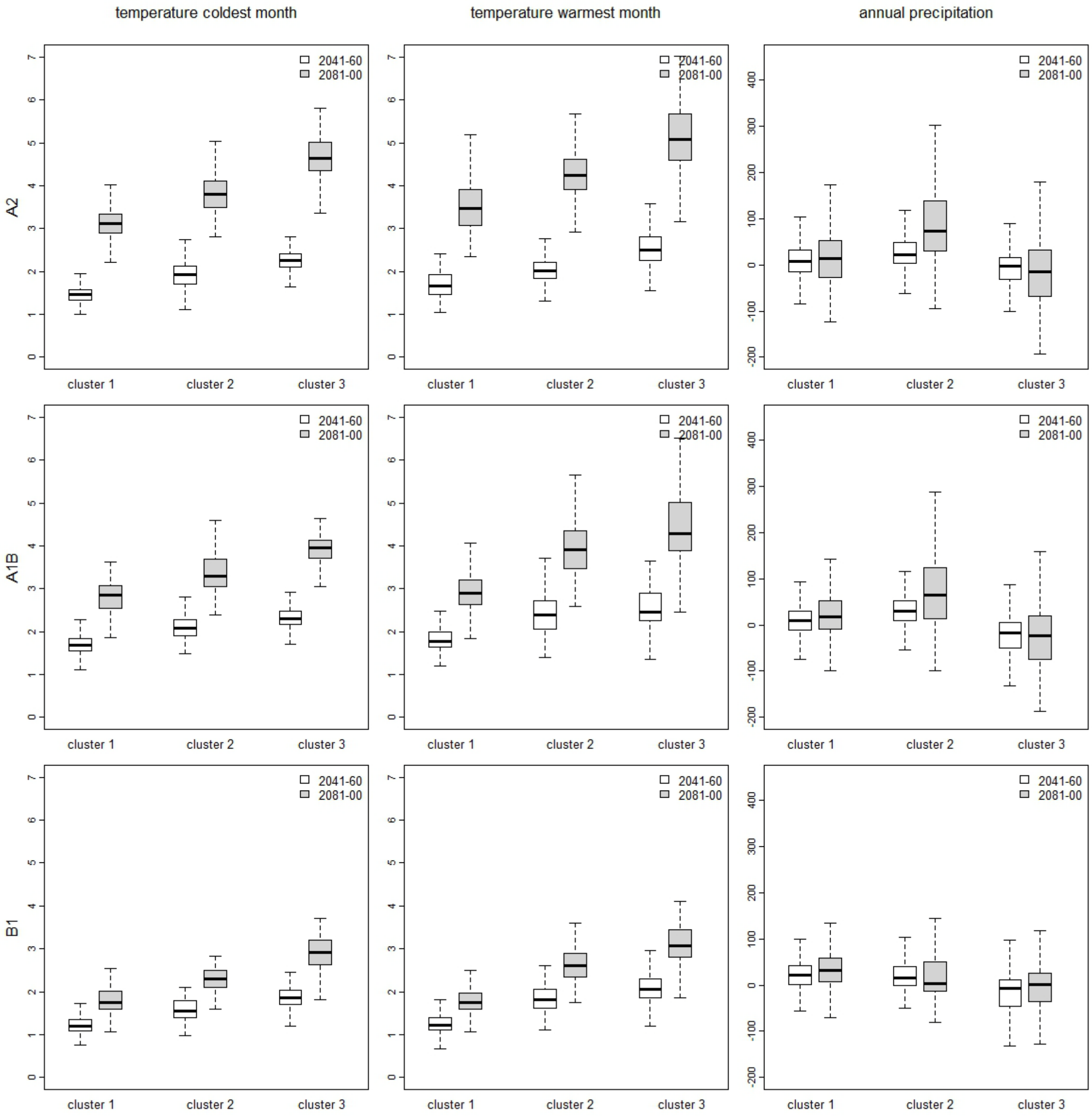

Supplement: Supplementary file 23 [file gcb0018-1253-SD12.pdf]
