# Supplementary material for: Exploring consensus in 21st century projections of climatically suitable areas for African vertebrates
Source: Glob Chang Biol. 2011 Dec 30;18(4):1253–69. doi: 10.1111/j.1365-2486.2011.02605.x (PMC3597255; doi:10.1111/j.1365-2486.2011.02605.x)

Appendix S13: Climate anomaly maps for the three variables

Difference between future (mid- and late-century) projections and baseline data for the three variables (annual precipitation and temperature of the coldest and warmest months), for the different General Circulation Model clusters (1 to 3) under alternative emissions scenarios (A2, A1B and B1). Note that for annual precipitation, the scale (in mm) covers both negative values (blue tones, referring to decreased precipitation in the future) and positive values (red tones, referring to increased precipitation in the future), whereas for the temperature-based variables the scale (in degrees Celsius) has positive values only (blue through to red tones indicating increasingly warmer future temperatures).

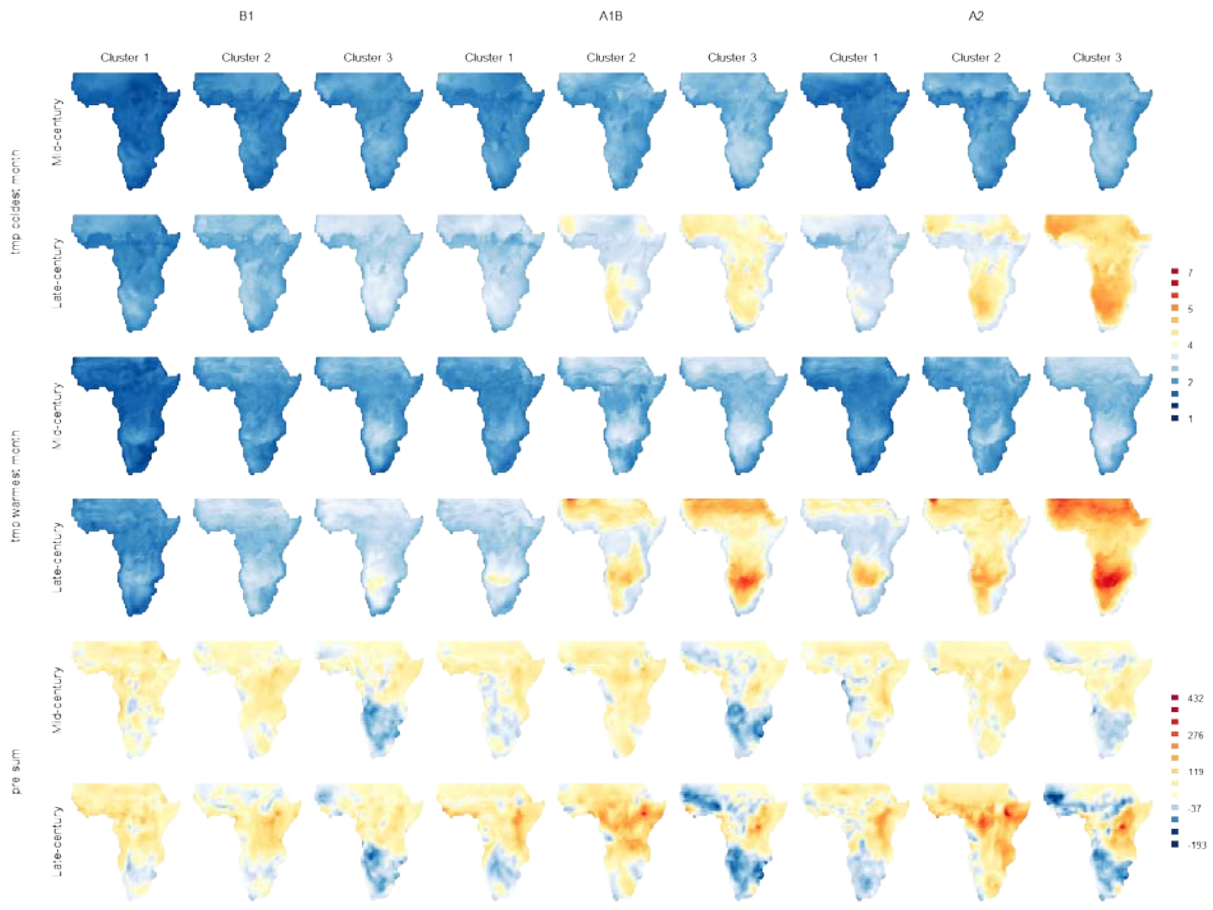

Supplement: Supplementary file 25 [file gcb0018-1253-SD13.pdf]
